# In utero rescue of neurological dysfunction in a mouse model of Wiedemann-Steiner syndrome

**DOI:** 10.1172/jci.insight.187039

**Published:** 2025-09-16

**Authors:** Tinna Reynisdottir, Kimberley J. Anderson, Katrin Möller, Stefán Pétursson, Andrew Brinn, Katheryn P. Franklin, Juan Ouyang, Asbjorg O. Snorradottir, Cathleen M. Lutz, Aamir R. Zuberi, Valerie B. DeLeon, Hans T. Bjornsson

**Affiliations:** 1 Louma G. Laboratory of Epigenetic Research, Faculty of Medicine, University of Iceland, Reykjavik, Iceland.; 2 Department of Genetics and Molecular Medicine, Landspitali University Hospital, Reykjavik, Iceland.; 3 Department of Anthropology, University of Florida, Gainesville, USA.; 4 Faculty of Medicine, University of Iceland, Reykjavik, Iceland.; 5 Department of Pathology, Landspitali University Hospital, Reykjavik, Iceland.; 6 The Jackson Laboratory, Bar Harbor, Maine, USA.; 7 McKusick-Nathans Departments of Genetic Medicine and Pediatrics, Johns Hopkins University, Baltimore, Maryland, USA.

**Keywords:** Genetics, Neuroscience, Epigenetics, Intellectual disability, Neurodevelopment

## Abstract

Wiedemann-Steiner syndrome (WDSTS) is a rare genetic cause of intellectual disability that is primarily caused by heterozygous loss-of-function variants in the gene encoding the histone lysine methyltransferase 2A (KMT2A). Prior studies have shown successful postnatal amelioration of disease phenotypes for Rett, Rubinstein-Taybi, and Kabuki syndromes, which are related Mendelian disorders of the epigenetic machinery. To explore whether the neurological phenotype in WDSTS is treatable in utero, we created a mouse model carrying a loss-of-function variant placed between 2 loxP sites. *Kmt2a^+/LSL^* mice demonstrated core features of WDSTS including growth retardation, craniofacial abnormalities, and hypertrichosis as well as hippocampal memory defects. The neurological phenotypes were rescued upon restoration of *KMT2A* in utero following breeding to a nestin-Cre. Together, our data provide a mouse model to explore the potential therapeutic window in WDSTS. Our work suggests that WDSTS has a window of opportunity extending at least until the midpoint of in utero development, making WDSTS an ideal candidate for future therapeutic strategies.

## Abstract 2

Genetic rescue of KMT2A during gestation rescues postnatal neurological dysfunction in a mouse model of Wiedemann-Steiner syndrome.

## Graphical abstract

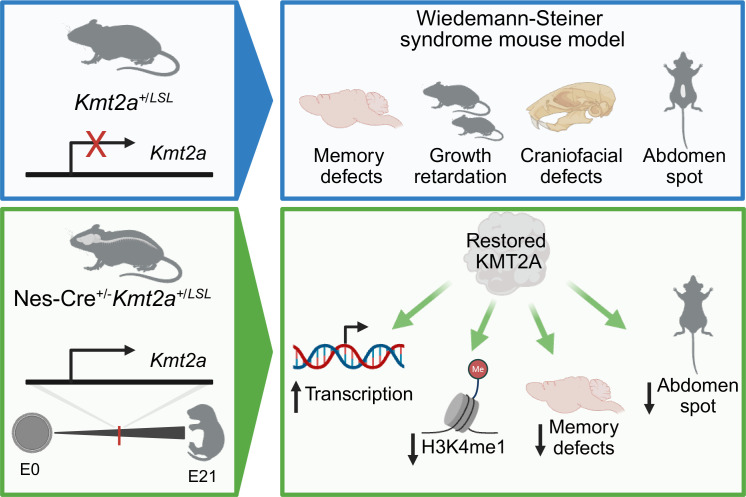

## Introduction

Wiedemann-Steiner syndrome (WDSTS, OMIM #605130) is a Mendelian disorder of the epigenetic machinery (MDEM), inherited in an autosomal dominant manner. WDSTS is primarily caused by de novo heterozygous variants in the gene encoding the histone-lysine methyltransferase 2A (*KMT2A*, previously *MLL*, *MLL1*, or *ALL* in the literature) (1). KMT2A plays a crucial role in the mono-, di-, and trimethylation of lysine 4 of histone 3 (H3K4me1/2/3) (2), modifications generally associated with active gene expression (3, 4), occurring at enhancers and promoters. KMT2A has been shown to be essential for postnatal neurogenesis in mice and as a regulator of neural progenitor proliferation and differentiation (5, 6). Key features of WDSTS include intellectual disability, growth retardation, hypotonia, craniofacial defects, gastrointestinal problems, and hypertrichosis. Previous mouse studies have shown postnatal rescue of neurological defects in several other MDEMs, including Rubinstein-Taybi (7), Rett (8), and Kabuki syndromes (9, 10). To extend these findings to WDSTS, we have created a mouse model for WDSTS (*Kmt2a^+/LSL^*). Prior mouse models have demonstrated that homozygous KO of *Kmt2a* (*Kmt2a^–/–^* mice) is lethal at birth, while heterozygous mice *(Kmt2a*^+/–^) exhibited growth retardation, skeletal defects, and hematopoietic abnormalities (11) as well as neurological defects in visuospatial and associative memory (12) and increased aggressive behavior (13). Notably, it has been shown that joint constitutive KO of the writer-eraser duo KMT2A and KDM5C rescues disease phenotypes seen in either disease model, including rescue of transcriptome abnormalities, neuronal structure, and aggressive behavior. This finding suggests that the enzymatic function of KMT2A plays a role in disease and that a specific balance is required among these histone machinery components (13, 14). In contrast, homozygous mice with a mutation specifically altering the C-terminal enzymatic SET domain remain viable and fertile (15). In concordance with the latter, our previous work has shown that missense variants causing WDSTS are mainly clustered outside of the enzymatic SET domain and within the DNA binding CXXC domain (16), suggesting that nonenzymatic roles of KMT2A may also be important in WDSTS pathogenesis.

To explore whether WDSTS is treatable in utero, we created and characterized a mouse model (*Kmt2a^+/LSL^*) that carries a heterozygous insertion of a loxP-stop-loxP (LSL) cassette in intron 1 of *Kmt2a* resulting in heterozygous KO of the affected allele. The model (*Kmt2a^+/LSL^*) serves as a disease model for WDSTS and allows temporal control of *Kmt2a*. By crossing the model to a nestin-Cre model, we demonstrate that in utero rescue of KMT2A levels could rescue WDSTS neurological phenotypes in primary neuronal progenitor cells (NPCs) from these mice and validated this finding by in vivo studies.

## Results

### Targeted KO of Kmt2a recapitulates key features of WDSTS.

WDSTS broadly affects patients with common clinical features within and outside the nervous system. To get an unbiased view of WDSTS phenotypes, we have summarized available data from an ongoing RARE-X WDSTS collection. The majority of the patients exhibit neurological issues (93.8%), most commonly intellectual disability and/or developmental delay, with a large part exhibiting behavioral and/or psychiatric problems (75.8%). Other common features include growth retardation (95.4%), dental and/or oral problems (79.4%), and hypotonia (60.0%). Distinctive features of WDSTS also include digestive system issues (76.9, most commonly constipation) and hypertrichosis cubiti (57%) (Table 1).

**Figure 1:**
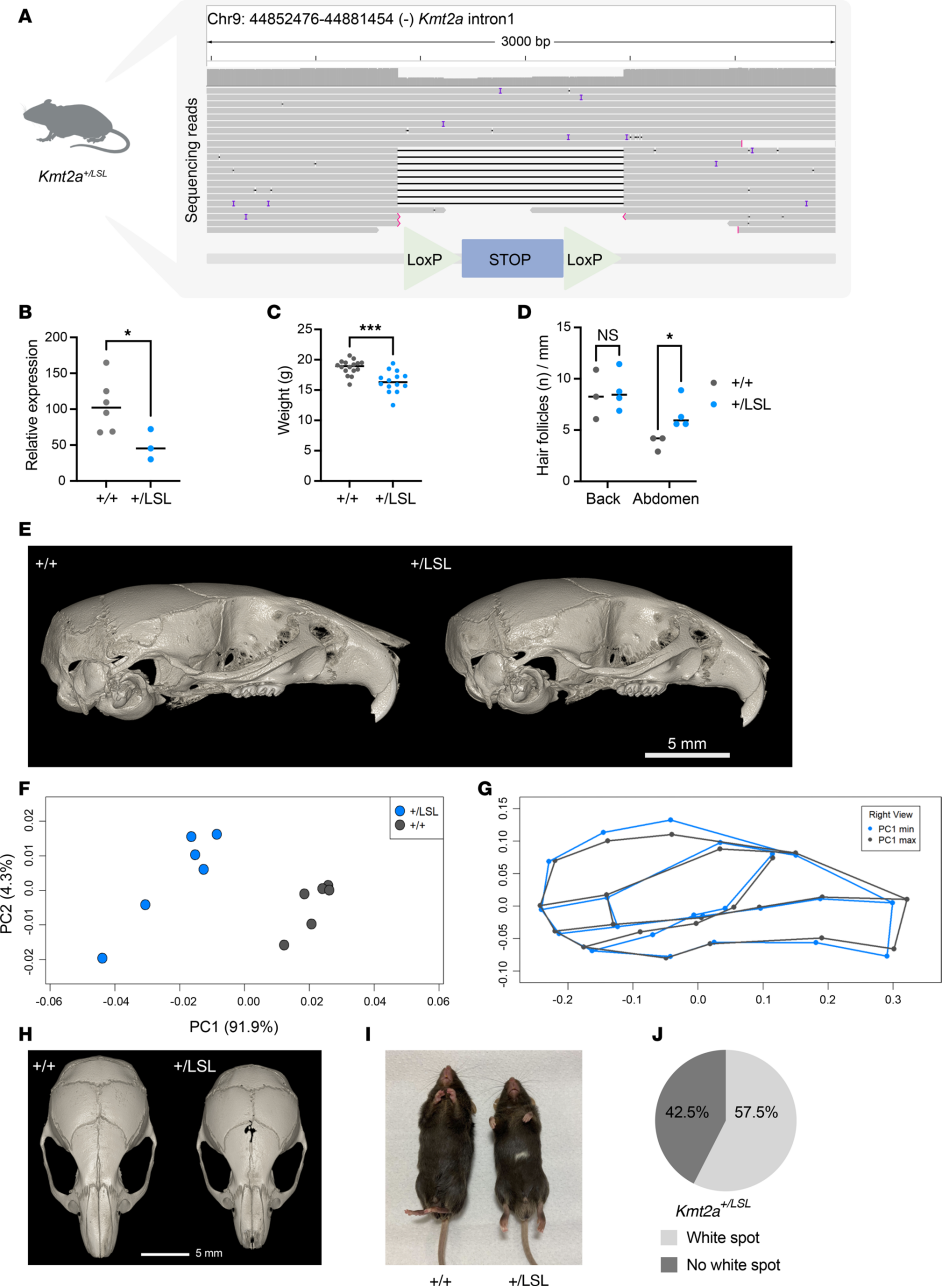
A Wiedemann-Steiner syndrome mouse model (*Kmt2a^+/LSL^*) recapitulates core phenotypes seen in individuals with WDSTS. (**A**) Schematic figure of *Kmt2a^+/LSL^* mouse model with a heterozygous loxP-stop-loxP cassette inserted in intron 1 of *Kmt2a* showing ONT sequencing reads of the cassette. Each line represents an individual long read including several that span the entire cassette as well as boundaries on either side allowing verification of its orientation. (**B**) Relative total *Kmt2a* mRNA expression is decreased in *Kmt2a^+/LSL^* mice compared with *Kmt2a^+/+^* littermates (P1, *n* = 7 *Kmt2a^+/+^* and *n* = 3 *Kmt2a^+/LSL^*, unpaired *t* test). (**C**) *Kmt2a^+/LSL^* mice weigh significantly less compared with *Kmt2a^+/+^* littermates (8-week-old, *n* = 24 *Kmt2a^+/+^* and *n* = 19 *Kmt2a^+/LSL^*, unpaired *t* test). (**D**) Hair follicle count per mm of skin is decreased on abdomen but not on the back of newborn *Kmt2a^+/LSL^* pups compared with littermates (P0, *n* = 3 *Kmt2a^+/+^* and *n* = 4 *Kmt2a^+/LSL^*, unpaired *t* test). (**E**) Representative CT images of *Kmt2a^+/+^* and *Kmt2a^+/LSL^* mice from lateral view, the depicting the craniofacial phenotype of 2- to 3.5-month-old mice. (**F**) PCA plot of the craniofacial structure showing a distinct separation of the genotypes along PC1 (*n* = 6 *Kmt2a^+/+^* and *n* = 6 *Kmt2a^+/LSL^*, 1-way Procrustes ANOVA). (**G**) The *Kmt2a^+/LSL^* mice are positioned at the negative end of PC1 and characterized by increased height and width of the neurocranium, a shortened midface, and ventral bowing of the cranium. (**H**) Representative figure of the gap within the interfrontal suture in the *Kmt2a^+/LSL^* mice (right) compared with wild type (left). (**I**) Representative figure of the white abdominal spot of a *Kmt2a^+/LSL^* mouse next to a WT littermate (2- to 3-month-old). (**J**) The majority of *Kmt2a^+/LSL^* mice (*n* = 40, 57.5%) present with a white abdominal spot, a phenotype not present in *Kmt2a^+/+^* littermates. *P < 0.05, **P < 0.01, ***P < 0.001, ****P < 0.0001.

To elucidate whether WDSTS shows in utero neurological malleability in response to genetic rescue, we created a mouse model (*Kmt2a^+/LSL^)* with a heterozygous LSL cassette insertion in intron 1 of *Kmt2a* (Figure 1A). The LSL cassette induces an early poly-A signal leading to immediate truncation of the mRNA transcript in intron 1 of the affected allele, allowing rescue of expression the of normal transcript with exposure to Cre-recombinase. The mouse model was created by the Jackson laboratory who tested functionality of the cassette mice by breeding *Kmt2a^+/LSL^* mice to a Sox2-Cre containing mouse model, which demonstrated that loxP sites were functional. To further verify the orientation and completeness of the cassette, we performed Oxford Nanopore Technologies (ONT) long-read sequencing on genomic DNA (gDNA) from *Kmt2a^+/LSL^* mice, from which single reads revealed the entirety of the cassette integrated into intron 1 of *Kmt2a* in the correct orientation (Figure 1A). Since this experiment provides whole-genome coverage, we also interrogated 2 predicted potential off-target sites from cassette integration and observe no obvious variants or indels at these sites (Supplemental Figure 1, A–D; supplemental material available online with this article; https://doi.org/10.1172/jci.insight.187039DS1). The offspring of the *Kmt2a^+/LSL^* mice show skewing from the expected Mendelian ratio with a significant bias against the inheritance of the mutant allele (*P* < 0.01, chi-squared test), with 40.9% (90 of 220) *Kmt2a^+/LSL^* mice born and 59.1% (130 of 220) *Kmt2a^+/+^* WT littermates. Similar to other *Kmt2a*-deficient models (11), *Kmt2a^+/LSL^* mice demonstrate lethality in homozygosity, with no *Kmt2a^LSL/LSL^* mice born (Supplemental Figure 1, E and F). As predicted, the *Kmt2a^+/LSL^* mice demonstrate ~50% reduction of *Kmt2a* mRNA transcripts as quantified by quantitative PCR (qPCR) (*n* = 3–7, unpaired *t* test, *P* < 0.05; Figure 1B). In addition to observing the appropriate integration of the LSL cassette, we observed characteristic WDSTS phenotypes of the *Kmt2a^+/LSL^* mice, supporting the notion that these mice adequately recapitulate the human disease. As previously reported in *Kmt2a^+/–^* mice (11), *Kmt2^a+/LSL^* mice exhibit growth deficiency with reduced weight compared with *Kmt2a^+/+^* littermates (unpaired *t* test, *P* < 0.001; Figure 1C and Supplemental Figure 2, A and B), alongside hemopoietic abnormalities (11), including a decreased lymphocyte percentage, an increase in monocyte percentages and an increase in platelet distribution width (Supplemental Figure 3). Given the consistent description of hypertrichosis in individuals with WDSTS (17), we decided to explore the skin histology of newborn P1 pups. *Kmt2a^+/LSL^* mice show an increased hair follicle count on the abdomen compared with their *Kmt2a^+/+^* littermates (unpaired *t* test, *P* < 0.05; Figure 1D), while there was no significant increase in hair follicle count on their back. Furthermore, we did not observe any obvious differences in hair regrowth upon removal of hair from the backs of the *Kmt2a^+/LSL^* mice compared with their WT littermates (Supplemental Figure 2C). This suggests that the basis of hypertrichosis in WDSTS involves an increased number of hair follicles rather than faster growth of individual hairs. Since severe constipation and other GI symptoms are common phenotypes in patients with WDSTS, we stained the distal colon sections of *Kmt2a^+/+^* and *Kmt2a^+/LSL^* mice for Calretinin, a clinical diagnostic marker for Hirschsprung’s disease (HD) that marks ganglion cells (18). Our results showed positive staining in both genotypes, indicating normal presence of ganglions and nerve fibers in our WDSTS model (Supplemental Figure 2D). Obviously, this does not rule out more subtle abnormalities of the enteric nervous system or problems relating to the function of the intestinal smooth muscle.

To evaluate whether craniofacial abnormalities are present, we performed CT scans on the *Kmt2a^+/LSL^* mice and their *Kmt2a^+/+^* littermates (*n* = 6 per genotype; Figure 1E). A 1-way Procrustes ANOVA yielded a significant effect of genotype on shape, and a principal components analysis (PCA) showed a clear separation between the groups along PC1, which describes 91.9% of the symmetric shape variance (*P* < 0.001, Figure 1F). Principal Components Analysis (PCA) showed a clear separation between the groups along PC1, which describes 91.9% of the symmetric shape variance (Figure 1F). The *Kmt2a^+/LSL^* mice show increased height and width of the neurocranium and ventral bowing of the cranium compared with littermates (Figure 1G). Additionally, we observed a prominent gap at the interfrontal suture in the *Kmt2a^+/LSL^* mice, which was not observed in any of the *Kmt2a^+/+^* littermates (Figure 1H). Similar craniofacial defects observed in a mouse model of Kabuki syndrome (9) have been linked to neural crest defects (19–21). Given the shared characteristics of these syndromes, it raises the question whether neural crest defects could also contribute to abnormalities found in WDSTS. Interestingly, in our model, a majority (57.5%) of the *Kmt2a^+/LSL^* mice present with a white abdominal spot, ranging from few distinct hairs to spots ~1-2 cm in length, most common along the midline (Figure 1, I and J, and Supplemental Figure 2E), suggestive of defects in the melanoblast migration from the neural crest (22). In contrast, we have never observed such a white spot in WT littermates.

### Observed neurological defects in the Kmt2a^+/LSL^ mouse model.

**Figure 2:**
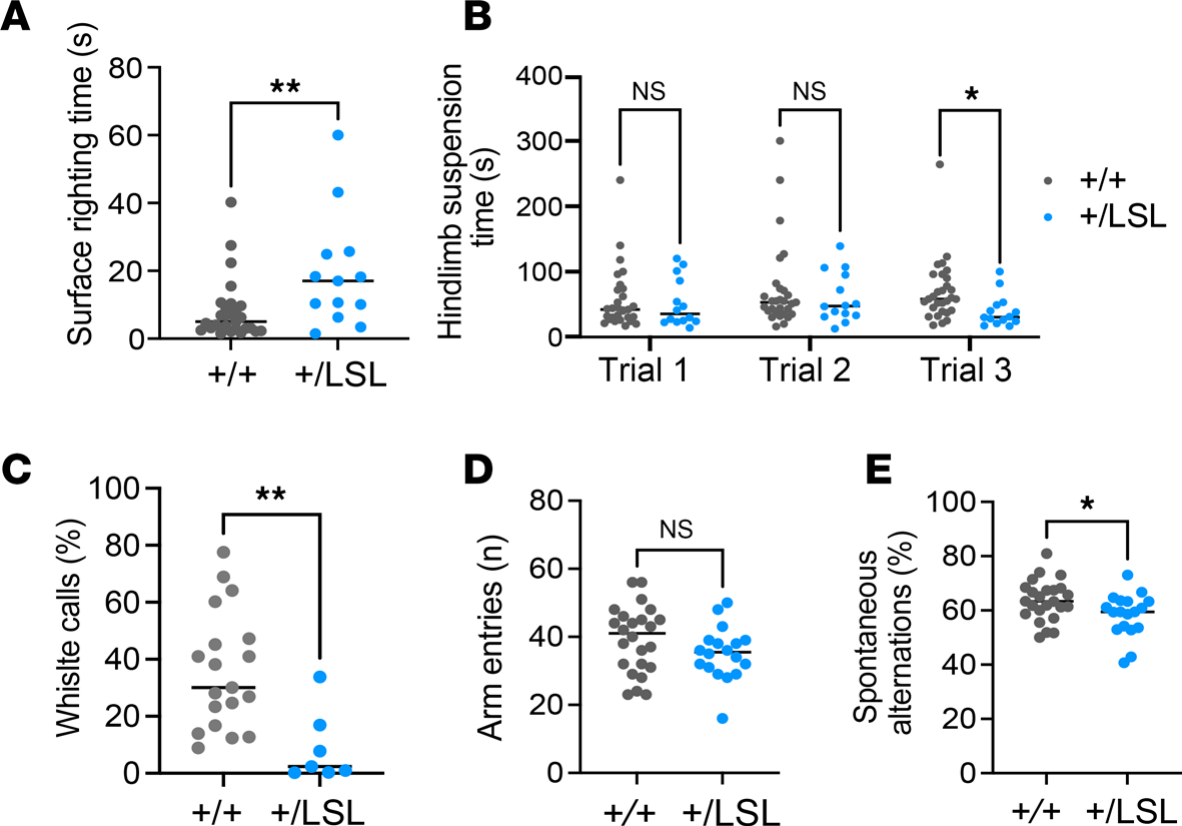
Behavioral abnormalities observed in the *Kmt2a^+/LSL^* mouse model. (**A**) Surface righting time in *Kmt2a^+/LSL^* mice compared with their WT littermates (P6, *n* = 27 *Kmt2a^+/+^* and *n* = 14 *Kmt2a^+/LSL^*). (**B**) Hindlimb suspension time in hypotonia tests for both genotypes (P6, *n* = 29 *Kmt2a^+/+^* *n* = 15, *Kmt2a^+/LSL^*). (**C**) Percentage of isolation-induced USV whistle calls for the *Kmt2a^+/+^* and *Kmt2a^+/LSL^* pups (P7, *n* = 5 *Kmt2a^+/+^*, *n* = 4, *Kmt2a^+/LSL^*). (**D**) Comparison of total arm entries in a Y-maze between adult *Kmt2a^+/LSL^* mice and their WT littermates (8- to 10-week-old, *n* = 24 for *Kmt2a^+/+^*, *n* = 18 for *Kmt2a^+/LSL^*). (**E**) Comparison of the percentage of spontaneous alternations between *Kmt2a^+/+^* and *Kmt2a^+/LSL^* mice (8- to 10-week-old, *n* = 24 for *Kmt2a^+/+^*, *n* = 18 for *Kmt2a^+/LSL^*). All significance levels were tested with an unpaired *t* test. *P < 0.05, **P < 0.01.

Consistent with the hypotonia observed in some patients with WDSTS, *Kmt2a^+/LSL^* mice exhibit significantly longer surface righting time compared with *Kmt2a^+/+^* littermates, indicating a lack of core strength and coordination (unpaired *t* test, *P* < 0.01; Figure 2A) and a significant decrease in hindlimb suspension time compared with *Kmt2a^+/+^*, indicative of increased fatigue in hindlimbs (unpaired *t* test, *P* < 0.05; Figure 2B), tested on P6 pups (*n* = 14–27). Next, we tested the social communication abilities of the pups by characterizing the isolation-induced ultrasonic vocalization (USV) of P7 pups when removed from the dam. Defects in whistle-like USVs by pups have been shown to be a consistent feature in mouse models of Autism spectrum disorder (ASD) (23–25). We found that the *Kmt2a^+/LSL^* pups showed a decreased percentage of isolation-induced USVs (*n* = 7 for *Kmt2a^+/LSL^* pups and *n* = 20 for *Kmt2a^+/+^* pups, unpaired *t* test, *P* < 0.01; Figure 2C), suggesting defects in their neurodevelopment consistent with an ASD phenotype. Assessing the anxiety-like behavior of adult mice in an open field test, we did not observe a significant difference in the time spent in center of the platform between genotypes (Supplemental Figure 2, F–H). The visuospatial memory function in adult *Kmt2a^+/LSL^* mice was tested in an open Y maze. The *Kmt2a^+/LSL^* mice did not exhibit a significant decrease in the number of arms entered during the test time, suggesting the adult mice do not exhibit a gross motor phenotype (unpaired *t* test, *P* = 0.1576; Figure 2D). However, the *Kmt2a^+/LSL^* mice demonstrate significant deficits in spontaneous alternations compared with *Kmt2a^+/+^* littermates, indicative of defective working memory reliant on the hippocampus and prefrontal cortex (*n* = 18–24 per genotype, unpaired *t* test, *P* < 0.05; Figure 2E) (26, 27). These findings are in concordance with previous studies describing the importance of *Kmt2a* for memory consolidation (12).

**Figure 3:**
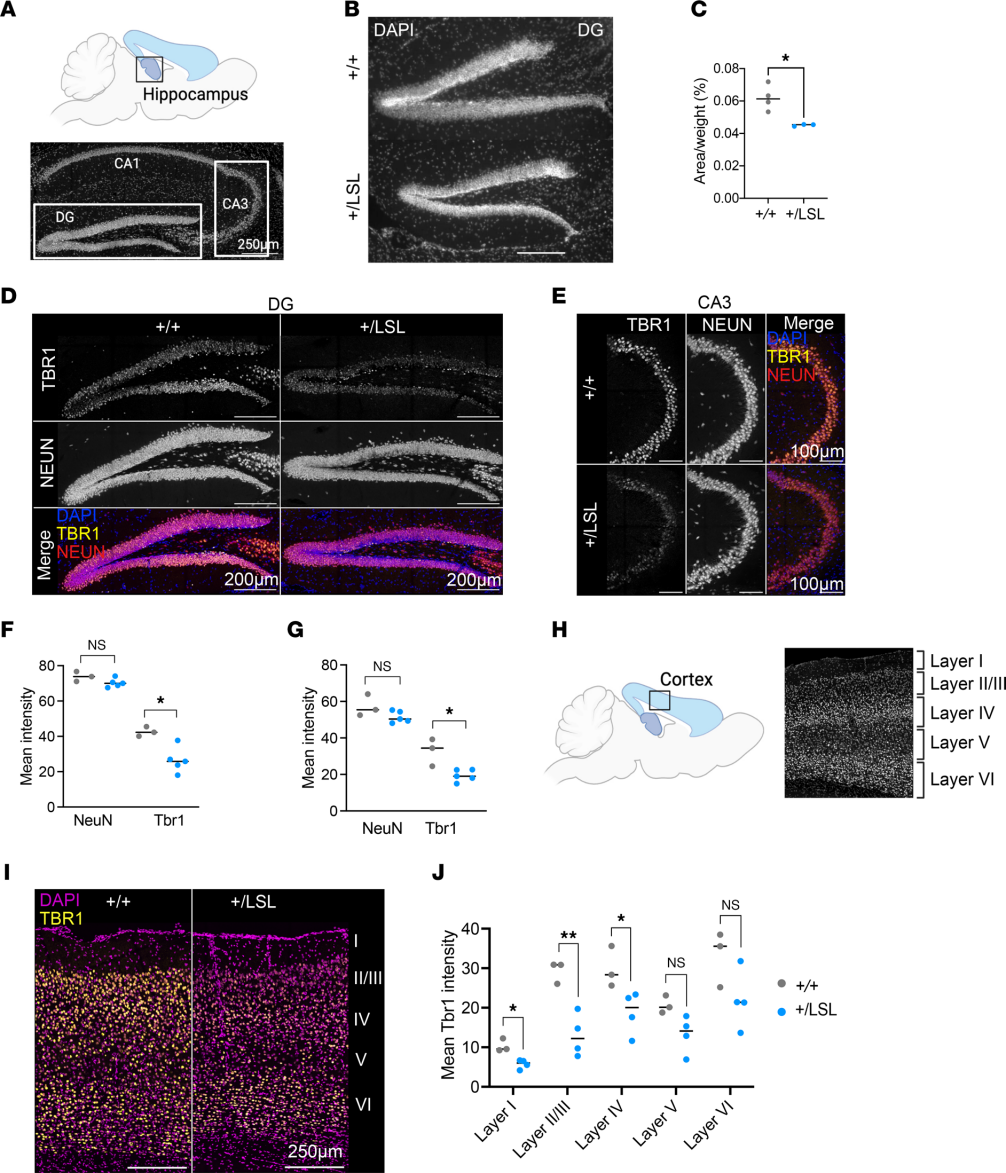
Neurological defects observed in the *Kmt2a^+/LSL^* mouse model. (**A**) Schematic figure of the adult mouse hippocampus with a representative image showing the DG, CA1, and CA3 regions of the hippocampus. All sections were performed on 9-week-old mice. Scale bar: 250 µm. (**B**) Representative images of DAPI staining of the DG area of hippocampus in *Kmt2a^+/+^* and *Kmt2a^+/LSL^* mice. Scale bar: 200 μm. (**C**) Quantification of granule cell layer area of the DG in *Kmt2a^+/LSL^* mice compared with WT littermates when corrected for body weight (*n* = 4 for *Kmt2a^+/+^*, *n* = 3 for *Kmt2a^+/LSL^* per genotype). (**D**) Representative NeuN and TBR1 staining of the DG area of the hippocampus in the *Kmt2a^+/+^* and *Kmt2a^+/LSL^* mice. Scale bar: 200 µm. (**E**) Representative NeuN and TBR1 staining of the CA3 area of the hippocampus in the *Kmt2a^+/+^* and *Kmt2a^+/LSL^* mice. Scale bar: 100 µm. (**F**) Quantification of NeuN and TBR1 levels in the DG (*n* = 3 for *Kmt2a^+/+^*, *n* = 5 for *Kmt2a^+/LSL^*). (**G**) Quantification of NeuN and TBR1 levels in the CA3 area (*n* = 3 for *Kmt2a^+/+^*, *n* = 5 for *Kmt2a^+/LSL^*). (**H**) Schematic of the adult mouse cortex location with representative images showing the layers of the cortex. (**I**) Representative images of DAPI and TBR1 staining in the cortical layers of the *Kmt2a^+/+^* and *Kmt2a^+/LSL^* mice. Scale bar: 250 μm. (**J**) Quantification of TBR1 levels in the cortical layers of *Kmt2a^+/+^* and *Kmt2a^+/LSL^* samples (*n* = 3 for *Kmt2a^+/+^*, *n* = 4 for *Kmt2a^+/LSL^*). All significance levels were tested with an unpaired *t* test. *P < 0.05, **P < 0.01, ***P < 0.001, ****P < 0.0001.

Next, we wanted to assess the structural phenotype of the neurological defects. First, we observed that the size of the granule cell layer of the dentate gyrus (DG) (Figure 3A), as measured as total size using DAPI staining, was significantly smaller in the *Kmt2a^+/LSL^* mice, compared with *Kmt2a^+/+^* littermates, when corrected for body weight (*n* = 3–4 per genotype, unpaired *t* test, *P* < 0.05; Figure 3, B and C). We note that the difference is not due to a smaller size of nuclei, as measured by average NeuN^+^ nuclei diameter (Supplemental Figure 4, A and B). A decreased size of the DG has been previously described in a mouse model of Kabuki syndrome (9) and later validated in individuals with Kabuki syndrome (28). To further investigate the neurological abnormalities in our *Kmt2a* model, we performed IHC staining of brain sections using antibodies against the neuronal marker NeuN and transcription factor TBR1 (Figure 3, D and E), verifying their specificity with 2°AB-only controls (Supplemental Figure 4C). TBR1 has been previously implicated in neurodevelopmental disorders, including ASD and ID (29). We observed that the *Kmt2a^+/LSL^* mice demonstrate significant reduction of *Tbr1* expression in the DG and CA3 of the hippocampus, compared with their WT littermates (unpaired *t* test, *P* < 0.05; Figure 3, F and G). However, the NeuN expression levels and cortical layer thickness ratios remained unchanged between genotypes (*n* = 3–5 per genotype, unpaired *t* test; Figure 3, F and G, and Supplemental Figure 4, D and E). Given the established role of TBR1 in neuronal migration and cortical layer formation (30), we investigated the TBR1 levels in the cortex (Figure 3H). While we observed the expected TBR1 staining pattern in the cortical layers of the control mice (31, 32) *Kmt2a^+/LSL^* mice exhibited decreased TBR1 levels in layers I–IV of the cortex, (*n* = 2–4 per genotype, unpaired *t* test, *P* < 0.05 for layer I/IV and *P* < 0.01 for layer II/III; Figure 3, I and J). Finally, we wanted to look further into the neurogenesis of the model and stained the brain sections for the mature granule cell marker Prox1 and migrating neuronal marker Doublecortin (DCX). Here, however, we observed no differences in the Prox1 expression levels nor the numbers of DCX^+^ cells, suggesting that there are not major problems in creating normal numbers of neurons in *Kmt2a^+/LSL^* mice (Supplemental Figure 4, F–I). Finally, we found no gross structural changes in the cerebellum of the *Kmt2a^+/LSL^* mice (Supplemental Figure 4J). Taken together, our findings support that the *Kmt2a^+/LSL^* mouse model recapitulates core human WDSTS disease phenotypes and allows for quantitative measurement of a number of metrics related to the neurological phenotype that can be used to investigate the in utero malleability.

### Nes-Cre^+/–^ Kmt2a^+/LSL^ mice exhibit successful cassette removal.

**Figure 4:**
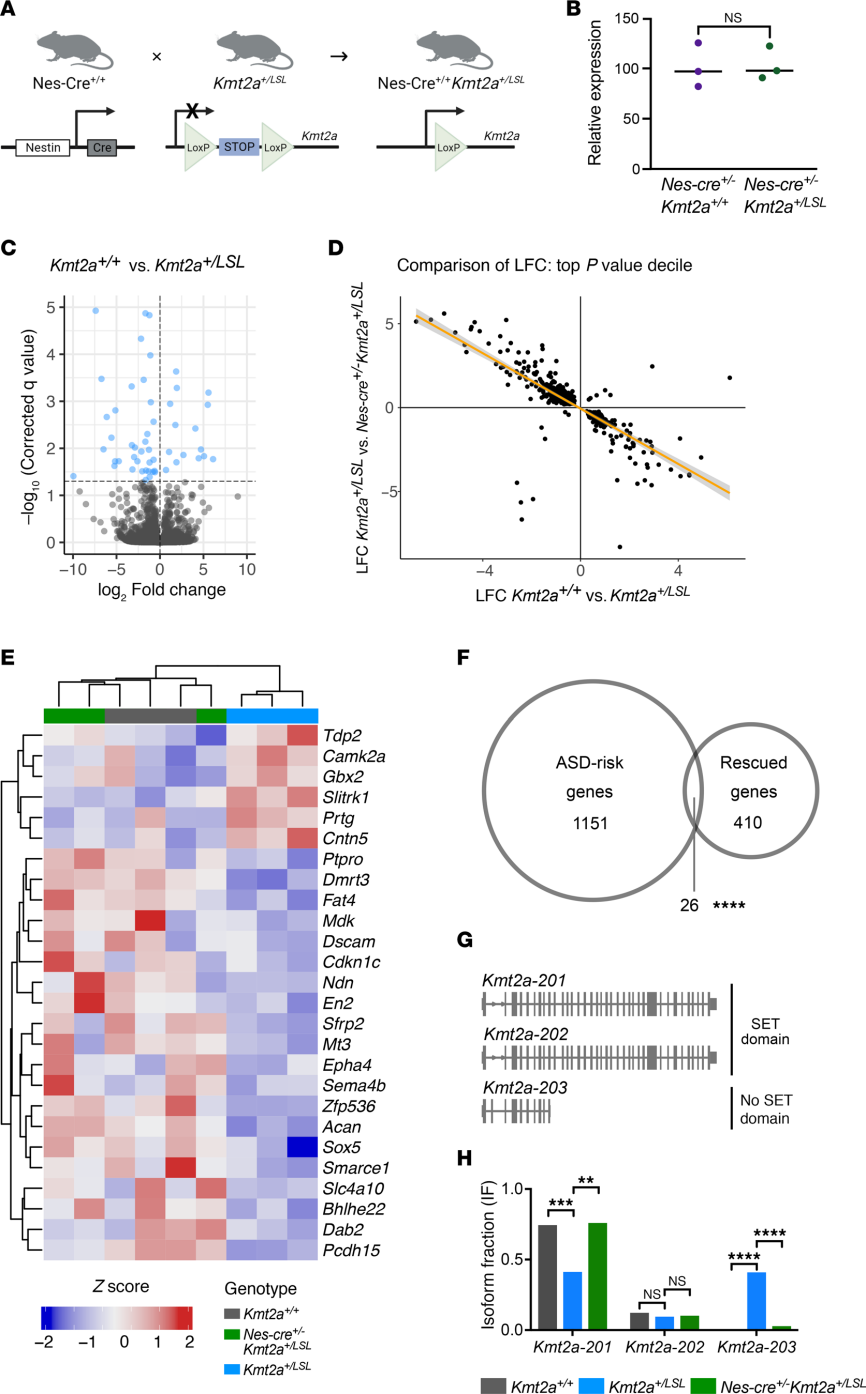
Transcriptional consequences of *Kmt2a* loss and rescue. (**A**) A schematic overview of the nestin-Cre strategy. By exposing the *Kmt2a^+/LSL^* mice to nestin-Cre, *Kmt2a* levels are expected to be restored in utero in the nervous system of the Nes-Cre^+/–^ *Kmt2a^+/LSL^* mice in utero, allowing us to test in utero rescue. (**B**) qPCR demonstrates that relative *Kmt2a* mRNA expression is unchanged in Nes-Cre^+/–^ *Kmt2a^+/LSL^* mice compared with Nes-Cre^+/+^*Kmt2a^+/+^* littermates in nervous tissue (P1, *n* = 3 Nes-Cre^+/–^ *Kmt2a^+/+^, n* = 3 Nes-Cre^+/–^ *Kmt2a^+/LSL^*, unpaired *t* test). (**C**) Volcano plot showing the log_2_ fold changes of differentially expressed genes comparing *Kmt2a^+/+^* versus *Kmt2a^+/LSL^* mNPCs (P0) with significant genes labeled in blue. (**D**) Correlation plot showing the negative correlation between log_2_ fold changes between *Kmt2a^+/+^* versus *Kmt2a^+/LSL^* and then between *Kmt2a^+/LSL^* versus Nes-Cre^+/–^ *Kmt2a^+/LSL^* mice (*R*^2^ = 0.57988). (**E**) Heatmap showing clustering of *Kmt2a^+/LSL^* samples compared with *Kmt2a^+/+^* and Nes-Cre^+/–^ *Kmt2a^+/LSL^* samples of the 26 rescued genes from significantly overrepresented biological pathways (with FDR < 0.05). Expression values were normalized to z-scores and plotted, with increased expression represented in red and decreased expression in blue. (**F**) Venn diagram of overlapping rescued genes with 1,177 known ASD-risk factor genes, with 26 genes overlapping (Fisher’s exact test). (**G**) Representative figure of *Kmt2a* isoforms and their exons. (**H**) Isoform switch analysis reveals a shift in isoform fraction (IF) in *Kmt2a^+/LSL^* mNPCs from the longer SET domain-containing *Kmt2a* transcript to the short non-SET domain containing *Kmt2a* transcript (GLM, FDR correction). **P < 0.01, ***P < 0.001, ****P < 0.0001.

To test in utero rescue of the neurological phenotypes, we crossed our *Kmt2a^+/LSL^* model with a nestin-Cre model (Nes-Cre^+/+^), which would be expected to lead to in utero rescue of *Kmt2a* expression in a cell type–specific manner in the nervous system at mid to late gestation (Figure 4A). We hypothesized that with a knock-in of *Kmt2a* expression in nervous tissue, the markers of neurological dysfunction would show rescue in Nes-Cre^+/–^ *Kmt2a^+/LSL^* mice, with a prior study (13) demonstrating rescue earlier, at the time of conception. To validate the efficacy of LSL cassette recombination in Nes-Cre^+/–^ *Kmt2a^+/LSL^* mice in the nervous system, we performed PCR on gDNA with primers targeting the LSL cassette using samples from the hippocampus and skin tissue from the ear and compared with Nes-Cre^+/–^ *Kmt2a^+/+^* controls. We observe an obvious decrease of the amount of LSL cassette in the nervous tissue but not in the skin tissue, of Nes-Cre^+/–^ *Kmt2a^+/LSL^* mice compared with controls (Supplemental Figure 5). To further validate the removal of the LSL cassette and the ability to regain normal *Kmt2a* expression, we quantified the relative mRNA expression in whole brain lysates of P1 pups and observed no significant difference between Nes-Cre^+/–^ *Kmt2a^+/+^* and Nes-Cre^+/–^ *Kmt2a^+/LSL^* mice (*n* = 3 per genotype; Figure 4B) in contrast to a significant difference between the genotypes without the Cre (Figure 1B).

### Gene expression abnormalities are rescued in Nes-Cre^+/–^ Kmt2a^+/LSL^ mNPCs.

To evaluate rescue of transcriptional abnormalities caused by heterozygous *Kmt2a*-deficiency in a homogenous cell population, we dissected out the hippocampus from newborn pups (P0) and cultured mouse NPCs (mNPCs). We performed RNA-Seq using mRNA from mNPCs from both genotypes to identify gene expression changes between *Kmt2a^+/+^* and *Kmt2a^+/LSL^* NPCs (*n* = 3 per genotype). We observed 64 significantly differentially expressed genes (DEGs) when comparing *Kmt2a^+/+^* and *Kmt2a^+/LSL^* samples with a majority of genes being downregulated (47 of 64 genes), in concordance with what is expected with the loss of a transcriptional activator (Figure 4C and Supplemental Table 1). Next, we wanted to assess whether there was an in utero reversal of the abnormalities we uncovered in the RNA-Seq dataset. First, we observe a significant overlap of 438 genes (Fisher’s exact test, *P* < 0.0001) within the top-decile of *P* values for comparisons between both *Kmt2a^+/+^* versus *Kmt2a^+/LSL^* and *Kmt2a^+/LSL^* versus Nes-Cre^+/–^ *Kmt2a^+/LSL^*. Secondly, there is a striking negative correlation of the log_2_-fold change (LFC) of these genes between the comparisons, where 96.9% of genes (423 of 438 genes) demonstrated such an effect (*R*^2^ = 0.57988; Figure 4D and Supplemental Table 2). In essence, these results indicate that downregulated genes from *Kmt2a^+/LSL^* versus *Kmt2a^+/+^* show upregulation in *Kmt2a^+/LSL^* mice in presence of the nestin-Cre and vice versa. We performed an overrepresentation analysis (ORA) of the genes showing such rescue and observed significant overrepresentation of pathways implicated in neuron development and neuron differentiation (Supplemental Figure 6A). We pooled the genes from the overrepresented pathways together and can observe a similar trend, with 20 of 26 genes downregulated in *Kmt2a^+/LSL^* samples (Figure 4E).

Notably, we observed downregulation of transcription factors, such as *En2* and *Sox5,* in *Kmt2a^+/LSL^* mNPCs compared with the *Kmt2a^+/+^* mNPCs. Disruption of these transcription factors has previously been linked to defects in hippocampal neurogenesis (33, 34), as well as being associated with intellectual disability and ASD (35, 36). Since up to 50% of patients with WDSTS have a diagnosis of ASD (37), we compared the rescued genes to known ASD-risk genes (38) and discovered a significant overlap of 26 genes (Fisher’s exact test, *P* < 0.0001; Figure 4F). Interestingly, *Kmt2a* was not among the significant DEGs in this dataset. However, upon further examination of individual exons in *Kmt2a^+/LSL^* cells, the majority of observed reads were limited to the first exon of *Kmt2a*, with an overall decrease in coverage for the full length *Kmt2a* transcript (Supplemental Figure 6, B and C). We then performed an isoform-specific analysis that revealed a significant shift of *Kmt2a* isoforms in the *Kmt2a^+/LSL^* samples, from the full-length canonical *Kmt2a-201* isoform to the truncated *Kmt2a-203* isoform, which only contains 9 of 36 of the *Kmt2a* canonical exons. This results in a product that solely has 4 of 15 KMT2A domains including 3 AT hooks and a CXXC domain but, more importantly, does not contain the enzymatic SET domain (Figure 4G). This shift is striking, with the *Kmt2a-203* isoform expression increasing from 0.076% in the *Kmt2a^+/+^* samples, to 40.2% in the *Kmt2a^+/LSL^* samples (Figure 4H). More notably, upon the LSL cassette removal in the Nes-Cre^+/–^ *Kmt2a^+/LSL^* cells, these effects are reversed, further confirming functionality of the cassette. Taken together, our findings support the notion that a heterozygous loss of *Kmt2a* leads to aberrant mRNA expression in mNPCs. These changes at the molecular level are then reversed in utero in the Nes-Cre^+/–^ *Kmt2a^+/LSL^* model.

### H3K4me1 signal is rescued but does not drive gene expression changes in Nes-Cre^+/–^ Kmt2a^+/LSL^ mNPCs.

**Figure 5:**
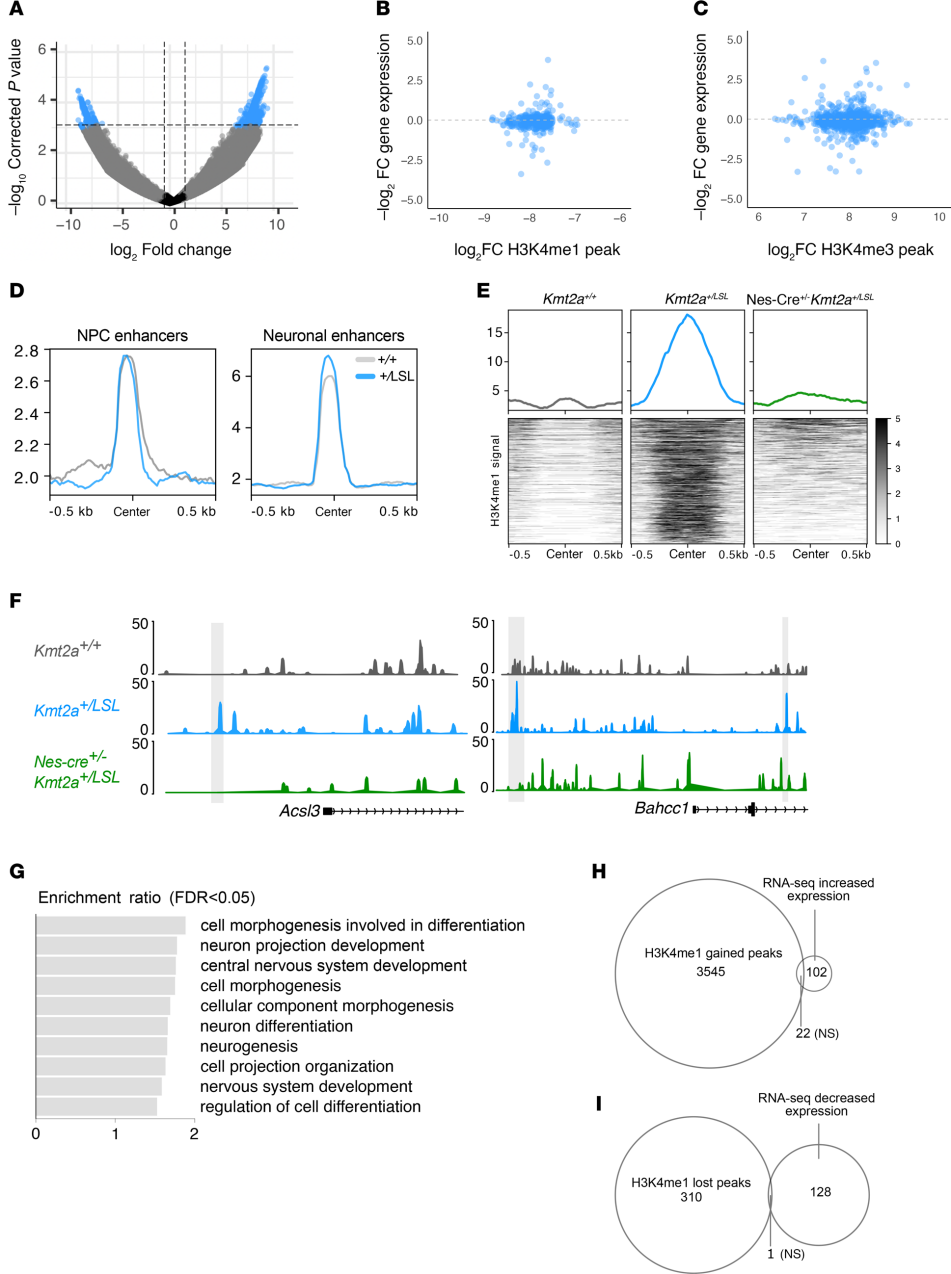
Epigenetic imbalance caused by heterozygous *Kmt2a* loss. (**A**) Volcano plot showing differential H3K4me1 peaks comparing *Kmt2a^+/+^* and *Kmt2a^+/LSL^* mNPC (P0) samples. (**B** and **C**) Expression changes for genes with lost H3K4me1 peaks (**B**) and gained H3K4me1 peaks (**C**) in *Kmt2a^+/LSL^* samples. (**D**) Signal plot of H3K4me1 signal for *Kmt2a^+/+^* and *Kmt2a^+/LSL^* samples over NPC and neuronal enhancers. (**E**) Heatmap of H3K4me1 signal at differential sites when comparing *Kmt2a^+/+^*, *Kmt2a^+/LSL^*, and Nes-Cre^+/–^ *Kmt2a^+/LSL^* samples. (**F**) Representative H3K4me1 signal figures, showing gained signal in *Kmt2a^+/LSL^* samples at neuronal enhancers (gray) at *Acsl3* and *Bahcc1* genes compared with WT and rescued samples. (**G**) Overrepresentation analysis of genes with differential H3K4me1 peak in *Kmt2a^+/LSL^* samples. Image shows enriched pathways (with FDR < 0.05). (**H**) Overlap of genes with a gained H3K4me1 peak and genes that have increased expression in *Kmt2a^+/LSL^* samples (Fisher’s exact test). (**I**) Overlap of genes with a lost H3K4me1 peak and genes that have decreased expression in *Kmt2a^+/LSL^* samples (Fisher’s exact test). *P < 0.05, **P < 0.01, ***P < 0.001, ****P < 0.0001.

To look further into the effects of heterozygous *Kmt2a* loss at the chromatin level, we performed a quantification of the H3K4me1 mark using CUT&RUN in mNPCs (*n* = 3 *Kmt2a^+/+^*, *n* = 4 *Kmt2a^+/LSL^* and Nes-Cre^+/–^ *Kmt2a^+/LSL^*). When comparing *Kmt2a^+/+^* and *Kmt2a^+/LSL^* samples, the majority of differential peaks (70.4%; 1,380 of 1,962 peaks) show a gain of H3K4me1 in *Kmt2a^+/LSL^* samples (Figure 5A and Supplemental Table 3). While mapping enhancer regions to genes is not trivial, when we linked the disrupted peaks to the nearest gene by distance, we found no correlation between the gene expression changes and the H3K4me1 epigenetic mark (Figure 5, B and C). Investigating the increased H3K4me1 signal further, we compared the signal over mouse NPC enhancer and neural enhancer regions from EnhancerAtlas (39). This revealed an increased signal at the neuronal enhancers but not at the NPC enhancers for *Kmt2a^+/LSL^* samples (Figure 5D). Again, the increased signal does not correlate with the nearest gene’s expression changes (Supplemental Figure 7A). To assess the rescue of this epigenetic mark in Nes-Cre^+/–^ *Kmt2a^+/LSL^* samples, we observe a reversal of 98.0% of peaks (Supplemental Figure 7B), and when visualizing the overall H3K4me1 signal at the differential peaks on a heatmap, we observe an increased signal in *Kmt2a^+/LSL^* samples compared with *Kmt2a^+/+^* mice, which is then reduced in Nes-Cre^+/–^ *Kmt2a^+/LSL^* samples (Figure 5E). This effect can be more specifically visualized on tracks showing gained H3K4me1 signal at neuronal enhancers for genes *Acsl3* and *Bahcc1* for *Kmt2a^+/LSL^* samples (Figure 5F). When applying a more relaxed cut-off, the 4,963 peaks that fell into the top decile of *P* values when overlapping the results from *Kmt2a^+/LSL^* versus *Kmt2a^+/+^* samples and the *Kmt2a^+/LSL^* and Nes-Cre^+/–^ *Kmt2a^+/LSL^* samples, map to 3,646 individual genes. When performing an ORA of these genes, we observe pathways involved in CNS differentiation, development, and neurogenesis (Figure 5G), highlighting that the disruption is occurring at functionally relevant sites. When examining the genes with restored H3K4me1 signal and gene expression, we observe a nonsignificant overlap of genes with a gained H3K4me1 peak and increased expression in *Kmt2a^+/LSL^* samples (Figure 5H), as well as a nonsignificant overlap of genes with a lost H3K4me1 peak and decreased expression in *Kmt2a^+/LSL^* samples (Figure 5I). Collectively, we observe an increased H3K4me1 signal in *Kmt2a^+/LSL^* samples, albeit with no global effects on gene expression.

### WDSTS phenotypes are rescued in utero.

**Figure 6:**
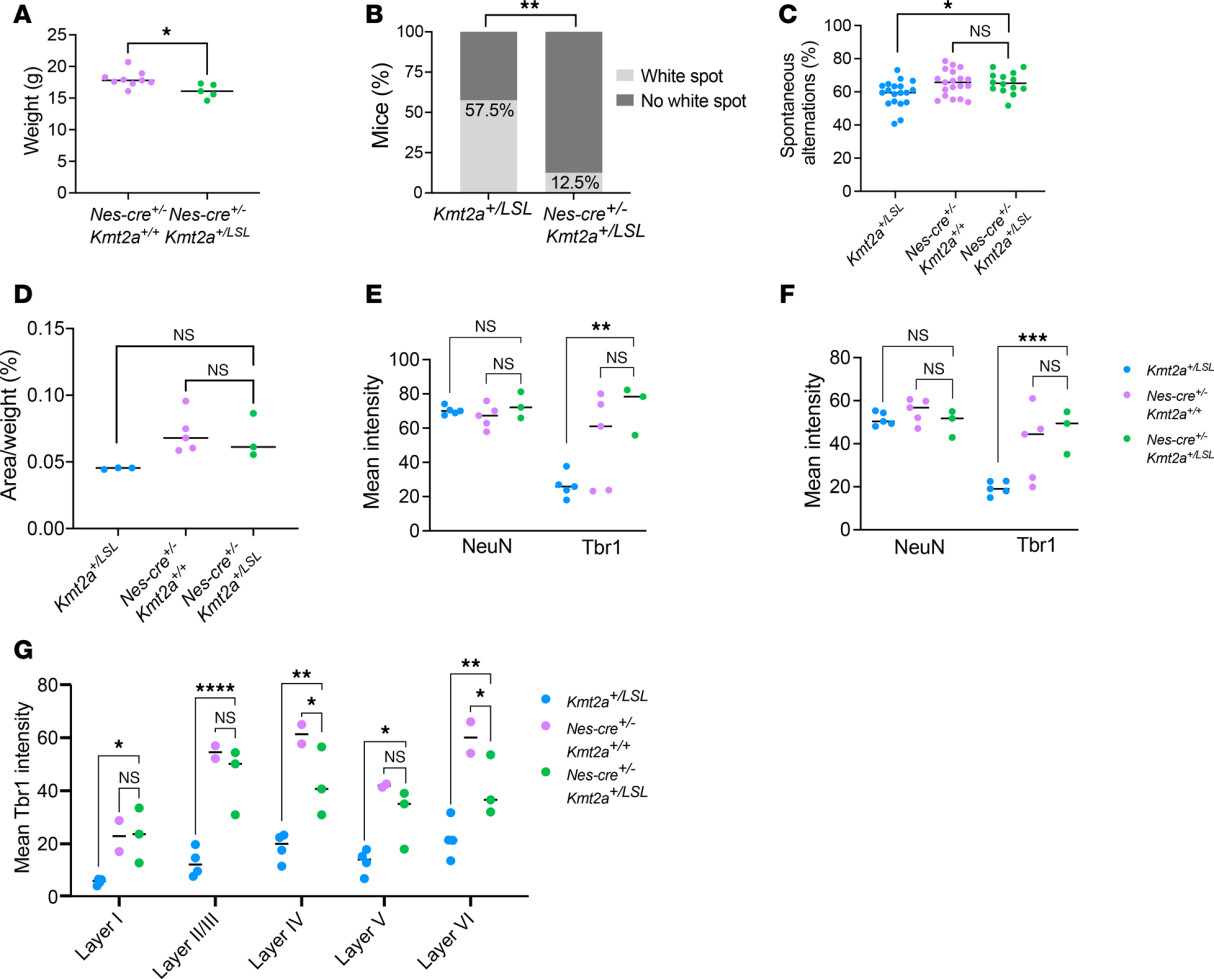
In utero rescue of neurological phenotypes in WDSTS mice. (**A**) Body weight of 8-week-old females for the Nes-Cre^+/–^ *Kmt2a^+/LSL^* mice compared with their Nes-Cre^+/–^ *Kmt2a^+/+^* littermates (*n* = 9 *Nes-Cre*^+/–^ *Kmt2a^+/+^*, *n* = 5 Nes-Cre^+/–^ *Kmt2a^+/LSL^*). (**B**) The presence of a white belly spot in Nes-Cre^+/–^ *Kmt2a^+/LSL^* mice (12.5%) compared with *Kmt2a^+/LS^* mice (57.5%, data from Figure 1J) (Fisher’s exact test). (**C**) Percentage of spontaneous alternations in the Nes-Cre^+/–^ *Kmt2a^+/LSL^* mice compared with their Nes-Cre^+/–^ *Kmt2a^+/+^* littermates and *Kmt2a^+/LSL^* mice (8–10 weeks old, *n* = 19 Nes-Cre^+/–^ *Kmt2a^+/+^*, *n* = 14 Nes-Cre^+/–^ *Kmt2a^+/LSL^*, *n* = 19 for *Kmt2a^+/LSL^*; data from Figure 2E). One-way ANOVA with a Dunnett’s correction for multiple comparisons was used to calculate significance. (**D**) Quantification of the size of the granule cell layer area of the DG of the hippocampus corrected for body weight (9-week-old, *n* = 5 Nes-Cre^+/–^ *Kmt2a^+/+^*, *n* = 3 Nes-Cre^+/–^ *Kmt2a^+/LSL^*, *n* = 3 *Kmt2a^+/LSL^*; data from Figure 3C). One-way ANOVA with a Dunnett’s correction for multiple comparisons was used to calculate significance. (**E**) Quantification of NeuN and TBR1 levels in the DG (9-week-old, *n* = 3–5 per genotype, *Kmt2a^+/LSL^*; data from Figure 3F). (**F**) Quantification of NeuN and TBR1 levels in the CA3 area of the hippocampus (9-week-old, *n* = 5 Nes-Cre^+/–^ *Kmt2a^+/+^*, *n* = 3 Nes-Cre^+/–^ *Kmt2a^+/LSL^*, *n* = 5 *Kmt2a^+/LSL^*; data from Figure 3G). (**G**) Quantification of TBR1 levels in the in cortical layers (9-week-old, *n* = 2 Nes-Cre^+/–^ *Kmt2a^+/+^*, *n* = 3 Nes-Cre^+/–^ *Kmt2a^+/LSL^*, *n* = 4 *Kmt2a^+/LSL^*; data from Figure 3J). For **E**–**G**, a 2-way ANOVA with a Dunnett’s correction for multiple comparisons was used to calculate significance. *P < 0.05, **P < 0.01, ***P < 0.001, ****P < 0.0001.

After demonstrating that nestin-Cre leads to sufficient recombination of the LSL cassette and rescued *Kmt2a* expression (Figure 4 and Supplemental Figure 5) we characterized the Nes-Cre^+/–^ *Kmt2a^+/LSL^* mice. The Nes-Cre^+/–^ *Kmt2a^+/LSL^* mice demonstrate a growth deficiency compared with their Nes-Cre^+/–^ *Kmt2a^+/+^* littermates (*n* = 5–9; Figure 6A and Supplemental Figure 8, A and B). Furthermore, we found similar craniofacial defects of the Nes-Cre^+/–^ *Kmt2a^+/LSL^* as previously described in *Kmt2a^+/LSL^* mice (Figure 1, E–H), indicating that there is no rescue of this phenotype (Supplemental Figure 8, C and D). This would suggest that either the growth defects of the *Kmt2a^+/LSL^* mice are not solely driven by nestin-expressing cell types or that the cause of the growth phenotypes occurs prior to when the rescue of *Kmt2a* takes place in utero. In contrast, the prevalence of the white abdominal spot of the Nes-Cre^+/–^ *Kmt2a^+/LSL^* mice drastically decreased from 57.5% to 12.5% compared with the 57.5% of the *Kmt2a^+/LSL^* mice, indicating malleability of this phenotype (Fisher’s exact test, *P* < 0.01; Figure 6B). Behavioral testing of adult mice demonstrates no difference of arm entries between Nes-Cre^+/–^ *Kmt2a^+/+^* and Nes-Cre^+/–^ *Kmt2a^+/LSL^* (*n* = 14-19, unpaired *t* test; Supplemental Figure 8E), nor of the percentage of spontaneous alterations in the Y-maze (*n* = 14–19, 1-way ANOVA with Dunnett’s correction; Figure 6C), indicative of nonimpaired working memory of Nes-Cre^+/–^ *Kmt2a^+/LSL^* mice. Similarly, the brain histology of the Nes-Cre^+/–^ *Kmt2a^+/LSL^* mice demonstrate normal hippocampal structure, with a nonsignificant difference in the size of the area of the granule layer of the DG, when compared with Nes-Cre^+/–^ *Kmt2a^+/+^* littermates and corrected for body weight (1-way ANOVA with Dunnett’s correction; Figure 6D). Furthermore, we observe no significant difference in the TBR1 levels in the DG nor CA3 areas of the hippocampus in Nes-Cre^+/–^ *Kmt2a^+/LSL^* mice compared with Nes-Cre^+/–^ *Kmt2a^+/+^* littermates (2-way ANOVA with Dunnett’s correction; Figure 6, E and F). While NeuN levels are consistent between all genotypes, we do observe a significant increase in the TBR1 intensity when compared with the *Kmt2a^+/LSL^* mice (*n* = 3–5 per genotype, 2-way ANOVA with Dunnett’s correction; Figure 6, E and F). Similarly, for the cortical layers, we find no or reduced significant difference between the Nes-Cre^+/–^ *Kmt2a^+/+^* mice and Nes-Cre^+/–^ *Kmt2a^+/LSL^*, but when compared with the *Kmt2a^+/LSL^* samples from Figure 3, we find a significant increase in the TBR1 intensity (2-way ANOVA with Dunnett’s correction; Figure 6G). Taken together, when bred to a nestin-Cre mouse model, we observe histological and behavioral rescue of the neurological defects of the Nes-Cre^+/–^ *Kmt2a^+/LSL^* mice.

## Discussion

In this paper, we have created and characterized a WDSTS mouse model (*Kmt2a^+/LSL^*). This model depicts many of the human WDSTS phenotypes, validating it as a disease model for WDSTS. We observed rescue of the neurological phenotypes of the mouse model after crossing the mice to a nestin-Cre model, demonstrating in utero malleability of WDSTS. Genetic disorders are being diagnosed earlier than before with the onset of whole genome sequencing and exome sequencing (40) and many strategies have successfully made it to the clinic including small molecules, ASO-based strategies and CRISPR-Cas9 based strategies (41, 42). A genetics-based approach assessing likelihood, therapeutic window, and phenotype plasticity can be valuable in prioritizing Mendelian disorders and providing genetic controls. Our work uses one such strategy for WDSTS, which could be extended to other congenital disorders. We demonstrate rescue of the altered *Kmt2a* allele and its consequences on multiple levels: genetic, molecular, and phenotypic.

Our work also provides temporal insights into the timing of disease onset in the CNS and neural crest. There is some debate in the literature surrounding the exact timing of action in the nestin-Cre mouse model, with the original paper suggesting recombination occurring as early as E11 in the nervous system (43, 44). However, more recent studies have shown that Cre expression only reaches sufficiently high levels in NSCs and NPCs to cause recombination during late embryonic (E15.5–E17.5) and early postnatal periods (43).

In vitro, *Kmt2a* loss downregulates most genes and rescue restores expression. In contrast, chromatin rescue runs opposite to expectations for H3K4 methyltransferase loss; the most affected sites gain H3K4me1 in *Kmt2a^+/LSL^* and normalize with rescue. Although the rescue is not complete, we think that the subtle differences at individual binding sites upon restoration of *Kmt2a* could be explained by the incompleteness of the rescue. A similar chromatin effect, with a trend toward increased chromatin accessibility, has previously been observed in studies of patients and animal models of Kabuki syndromes (45, 46) and in cells from a mouse model of Kleefstra syndrome (47). Moreover, the increased H3K4me1 signal at neuronal enhancers could suggest the cells are priming these enhancers toward a premature neuronal phenotype, similar to what has been described in Kabuki syndrome (48, 49). Another potential reason for this observation could be that the increased H3K4 methylation involves a compensatory response. We also observe signs of increased transcriptional initiation of *Kmt2a* and isoform switching in *Kmt2a^+/LSL^* samples, showing that attempted compensation is taking place at least at the locus itself. However, of the enzymes known to affect the H3K4 substrate, none show significant differential expression between the 2 genotypes in our RNA-Seq data, so this notion would need further validation. Alternatively, we hypothesize that KMT2A might be occupying binding sites, effectively blocking other epigenetic or transcriptional factors from binding at those loci. Gene-expression changes do not correlate with H3K4me1 changes, indicating that KMT2A catalytic loss is not the sole driver of WDSTS. This is consistent with prior data showing WDSTS missense variants are not enriched in the KMT2A SET domain, unlike Kabuki variants clustered in KMT2D’s SET domain (16, 50). This is emphasized even further with the fact that homozygous *Kmt2a* catalytic-domain deletion yields viable and fertile mice while such deletions lead to lethality in other *Kmt2* mouse models — e.g., *Kmt2d* (9, 11). Further studies will be needed to understand the underlying mechanism of this effect in more detail.

In addition to previously described features, the *Kmt2a^+/LSL^* model demonstrates multiple additional common WDSTS-like phenotypes such as craniofacial defects, hypotonia, and hypertrichosis, one of the defining features of WDSTS (Supplemental Table 1). Interestingly, our mice show increased hair follicle density in some regions, without an obvious difference in the rate of regrowth of the hair, and could therefore open an avenue of research into the mechanistic basis of hypertrichosis in humans. We observe no rescue of the growth retardation of Nes-Cre^+/–^ *Kmt2a^+/LSL^* mice. However, it is important to note that the nestin-Cre mice have previously been shown to demonstrate mild hypopituitarism, accompanied by decreased body weight, and to ensure that these results could not be due to the nestin-Cre genotype, we compared their weight to the Nes-Cre^+/–^ *Kmt2a^+/+^* littermates (51). Similarly, we observed no rescue of the craniofacial structure abnormalities in the Nes-Cre^+/–^ *Kmt2a^+/LSL^* mice. This could be explained in 2 ways. Either the growth retardation might be related to dysfunction in cell populations that do not express nestin, or secondly, the growth retardation might occur early enough in development to not be rescued by the nestin-Cre expression. In both scenarios we would not expect a rescue of these phenotypes. Finally, we observed decreased TBR1 expression in the brains of *Kmt2a*-deficient mice. While loss of TBR1 is a known contributor to cortical malformations and intellectual disability (29), its specific role in the pathogenesis of WDSTS remains unclear. Further studies will be necessary to elucidate the mechanistic contribution of this alteration to WSS.

Previous studies have linked abnormal craniofacial features to neural crest dysfunction in KS in mice (19–21), a prominent phenotype shared between KS and WDSTS. In concordance, we observe a prominent white abdominal spot in the majority of *Kmt2a^+/LSL^* mice, with a striking decrease in the Nes-Cre^+/–^ *Kmt2a^+/LSL^* mice, without fully eliminating it. This could suggest the timing of rescue of the neural crest defects is around the same time as nestin expression starts, showing partial rescue in our model. Ultimately, our findings suggest a role for *Kmt2a* in neural crest development with more research needed to fully elucidate its role. Lastly, we observe rescue of the neurological dysfunction in the Nes-Cre^+/–^ *Kmt2a^+/LSL^* mice both histologically and behaviorally. However, a limitation of the present study is that we did not test rescue using a postnatal-specific Cre. This leaves 2 major questions unresolved: the feasibility of postnatal rescue and the upper limit of the therapeutic window in WDSTS. Future studies are needed to address these questions.

We conclude the neurological phenotype of WDSTS is malleable in utero and that WDSTS is yet another potentially treatable cause of ID from the group of Mendelian disorders of the epigenetic machinery.

## Methods

### Sex as a biological variable.

Our study examined male and female animals, and similar findings are reported for both sexes.

### Mouse models.

The mouse model was created by the Jackson Laboratory (Bar Harbor, Maine, USA), by microinjection of C57BL/6J single cell zygotes with CRISPR/Cas9 using guides 5′-CTGCAGCGAGAGACTGTATG-3′ and 5′GAGACTGTATGAGGTATCAG-3′ with a donor plasmid containing a lox-STOP-lox element flanked by *Kmt2a* intron 1 genomic DNA sequences of 1.4 kb and 1.8 kb, respectively. Of 38 mice recovered, 6 founders were identified with the desired genome edited allele at the *Kmt2a* locus, as determined by genomic long-range PCR. Two lines were established after mating of the founders to C57BL/6J mice to confirm germ line transmission of the edited lox-STOP-lox knock-in (KI) allele in *Kmt2a* and subsequently expanded by an additional backcross to C57BL/6J mice (JAX Stock #664) prior to intercrossing and strain validation (52). These 2 conditional KI (cKI) lines, designated as stock numbers #35724 C57BL/6J-*Kmt2a^em7Lutzy^*/J and #37525 C57BL/6J-*Kmt2a^em8Lutzy^*/J each contain the same allele but were derived from different founders. The functionality of the loxP elements within each KI allele was confirmed by test mating of heterozygous #35724 and #35725 male mice with hemizygous B6.Cg-*Edil3^Tg(Sox2–cre)1Amc^*/J female Cre-expressing mice (JAX Stock #8454) (53). Whereas both strains were mated with Sox2-Cre to confirm loxP functions, postCre Het x Het matings were only performed using the em7.1 allele (JAX Stock #35727, now extinct). Sox2-Cre Tg did not segregate in these mice (Supplemental Figure 1G). This validation revealed both stocks #35724 and #37525 behaved the same way in subsequent matings and therefore only Stock #35724, named *Kmt2a^+/LSL^*, was characterized further with respect to expression profiling in the pre- and post-Cre mice. Jackson Laboratory Stock #35724 can be purchased from the Jackson Laboratory mouse mutant repository (https://mice.jax.org). Genotyping was performed from DNA isolated from ear clips using primers Kmt2a-genoF1 (5′-ACA CAT GGC TTC CTG GAG G-3′) and Kmt2a-genoR1 (5′-CTG TTT GCA GTC GGA AAG CC-3′). Nestin-Cre^+/+^ mice (JAX stock #3771) were acquired from the Jackson Laboratory (54, 55). Nestin-Cre model was genotyped using primers NesCre-WT-F-(5′-TTG CTA AAG CGC TAC ATA GGA -3′), NesCre-Tg-F-(5′-CCT TCC TGA AGC AGT AGA GCA-3′), NesCre-R-(5′- GCC TTA TTG TGG AAG GAC TG-3′). All mouse strains were maintained on pure C57BL/6J background and provided food (Altromin NIH#31 M (breeding) or Altromin 1324 (maintenance), Brogaarden) and filtered water ad libitum. The animals were maintained under a 12-hour light/dark cycle at room temperature (21°C–23°C) with relative humidity around 40%. Regular FELASA health monitoring was conducted with samples taken from sentinel and resident animals and analyzed by IDEXX Germany, yielding negative results during experimental period.

### Behavioral testing.

The hypotonia tests (surface righting and hindlimb suspension tests) were performed on P6 pups as described by Feather-Schussler et al. (56). For the surface righting test, the pups were placed on their backs and held in place for 5 seconds, recording the time for the pups to return to a prone position. The test was conducted in 3 trials, giving the pup a 30 second rest in between each trial and a time-out at 60 seconds, with mean scores over all trials plotted. For the hindlimb suspension test, the pups were placed on their hindlimbs on the rim of a 50 mL conical tube, with the latency to fall recorded. The test was conducted in 3 trials, giving the pup a 60-second rest in between each trial and a time-out at 5 minutes. All trials were plotted, with each trial tiring the mice more. We tested isolation-induced USV in P7 pups by placing the pups in a soundproof chamber and recording their USVs for 4 minutes. The USVs were analyzed using the UltraVox XT (version 3.2) software. The open field testing was performed on 8-week-old mice by placing them in a 30x30 cm box which they could freely explore for 10 minutes. The data were analyzed using EthoVision XT software (Noldus, version 14). The spontaneous alternation Y-maze was performed on 8- to 10-week-old mice according to protocol described by Kraeuter et al. (57). Spontaneous alternation percentage is calculated with the following formula:

### CT scan.

CT scans were performed on 2- to 3.5-month-old mice (3-6 mice per genotype) using 50 kV tube voltage, 0.21 mA tube current and 1,712 mGy dose estimate using the pre-clinical optical imaging and CT system from MILabs (Houten, Netherlands). Virtual reconstructions of the skull were produced for each specimen by importing the CT data to 3dSlicer at half resolution (0.030 x 0.030 x 0.030 mm3 cubic voxels), and generating a model of the skull surface based on thresholding. A geometric morphometric approach allowed us to estimate cranial shape based on 3D coordinate data for a set of 31 biologically homologous landmarks (Supplemental Table 2) evenly distributed across the cranium. Landmark coordinate data were exported from 3dSlicer (58) and we used the R geomorph package (v.4.0.7) for all morphometric analyses (59). We assumed object symmetry for each specimen using the bilat.symmetry() function, which removes the effect of asymmetric variation (60). For a subset of our data, rostral and caudal ends of the crania were inadvertently truncated in the original CT scanning episode. This resulted in missing data for nasale (nal) in 6 specimens, and for opisthion (opi), basion (bas), and occipital condyles (rfmc and lfmc) in 11 specimens. Missing data were estimated using the estimate.missing() function, which predicts the location of those landmarks based on the average of other individuals in the sample (61). Both *Kmt2a^+/LSL^* and WT genotypes were equally represented in this average sample. For each analysis, we conducted a new Procrustes superimposition and tested the effect of genotype using a Procrustes ANOVA with the procD.lm() function. We visualized the results using Principal Components Analysis (PCA).

### Perfusion, cryosectioning, IF staining.

Mice were euthanized at 9 weeks old by injection of 500 µL of a Xylazine (4 mg/mL final concentration, Chanazine, Chanelle) and Ketamine (16 mg/mL concentration, Ketexx Vet) combination in 0.9% NaCl and perfused transcardially with 4% PFA after first flushing the blood system with 1x PBS (Santa Cruz Biotechnology Inc.). Brains were removed and cryopreserved in a 2-step gradient of 30% and 15% sucrose solution (sucrose [Invitrogen] in 0.1M phosphate buffer, pH 7.2). Brains were then cut sagittally and frozen in Tissue-Tek OCT compound. Brains were sectioned in 20μm serial sections on Leica CM1850 cryostat (Leica Biosystems). Sections were stored at –80°C until stained. Every second, sixth, tenth, and fifteenth brain section were used for DAPI staining. Samples were then washed 2x10 minutes with TBS-T (1x TBS [Santa Cruz, sc-24951] with 0.05% Triton X-100 [Sigma-Aldrich, T8787-100ML]) and then treated in sodium citrate buffer (10mM sodium citrate, 0.05% tween-20, pH 6) at 95°C for 20 minutes for antigen retrieval, washed 3x10 minutes in TBS-T and then blocked for an hour in TBS-T+ (TBS-T with 3% normal goat serum [Abcam, ab7481]) at room temperature. Samples were incubated in primary antibody solution (antibody in TBS-T+) overnight at 4°C. Slides were washed 3x10 minutes in TBST-T and mounted with Fluoromount-G with DAPI (Thermo Fisher Scientific, 00-4959-52). To estimate the size of the granule cell layer, we manually traced it using Fiji software. The student performing the tracing was blinded to the genotype. Fiji was then used to calculate the traced area, and the measurements were averaged across 4 slices per animal. Finally, we compared the granule cell layer size between individual animals, both with and without correction for body weight. Primary antibodies used Alexa Fluor 647 Anti-NeuN antibody (Abcam, ab190565 1:500 dilution). Brain samples were imaged using a CrestOptics CICERO spinning disk with a 10x and 40x objective.

### Staining of TBR1, NeuN, PROX1, MAP2, and DCX.

Sections were washed 3x in TBS, then subjected to heat antigen retrieval at 85°C for 20 minutes in sodium citrate buffer. The sections were then cooled gradually to room temperature and washed 1x in TBS. Sections were permeabilized for 20 minutes in 0.5% triton-X 100 in TBS, before being washed once with wash buffer (TBS with 0.05% triton-X 100). Samples were incubated for 1 hour at room temperature with blocking buffer (TBS with 0.05% triton-X 100 and 5% normal goat serum). Primary antibodies (Set 1: 1:200 Prox1, BioLegend #925201; 1:100 DCX E-6, Santa Cruz #sc-271390; Set 2: 1:300 TBR1 Abcam #ab183032; 1:200 MAP2, Abcam #ab11267) were diluted in blocking buffer, then added to sections and incubated overnight at 4°C in a humidity chamber. Slides were equilibrated to room temperature for 30 minutes, then washed 3x5 min in wash buffer. Secondary antibodies (Set 1: Goat Anti-Rabbit IgG H&L Alexa Fluor® 488, Abcam #ab150077, Goat anti-Mouse IgG (H+L) Secondary Antibody Alexa Fluor 555, Thermo #A32727; Set 2: Goat Anti-Rabbit IgG H&L Alexa Fluor® 488, Abcam #ab150077, Goat Anti-Mouse IgG H&L Alexa Fluor® 647, #ab150115) were diluted 1:1,000 in blocking buffer, before being added to sections and incubated for 1 hour at room temperature. Slides were washed 3x10 min with TBS, then incubated overnight at 4°C with primary conjugated antibodies (Set 1: 1:200 Alexa Fluor 647 Anti-NeuN antibody [EPR12763], Abcam #ab190565; Set 2: 1:200 MAP-2 Antibody (A-4) Alexa Fluor 594, Santa Cruz #sc-74421 AF594). Slides were washed 2x5 minutes with TBS, before being incubated for 20 minutes at room temperature with 1 μg/mL DAPI in TBS. Slides were washed 1x with TBS, before being coverslips were mounted with ProLong Gold Antifade Mountant (Thermo, #P36930). Slides were incubated overnight at room temperature in the dark, exposed to air, then stored at 4°C prior to imaging. Slides were imaged with an Olympus FV4000 confocal using 30x UPlanSApo 1.3NA Silicone objective, using a z-step size of 1.5 μm. Stitching was performed using the cellSens FluoView software. Fiji was used to generate maximum intensity projections and analysis (62). Each data point represents a biological replicate which is a calculated average of 2–5 technical replicates.

### Mouse NPC extraction and culture.

Mouse NPCs were dissected from hippocampus of P0 pups and cultured in an adapted protocol from Bernas et al. (63). Dissected hippocampi were dissociated in 1X TrypLE (Thermo Fisher Scientific, A1217701), washed in Neurobasal growth medium: Neurobasal medium (Thermo Fisher Scientific, 21103049) with 1X B27 supplement (Thermo Fisher Scientific, 17504044), and filtered through a 70 μm cell strainer (Miltenyi Biotech, 130-110-916). The cell suspension was centrifuged at 350g for 5 minutes, resuspended in 500 μL Neurobasal growth medium containing 1X Penicillin/Streptomycin (Sigma, P4333-100ML), 1X Glutamax (Thermo Fisher Scientific, 35050038), 20ng/mL FGF-2 (Peprotech, 100-18B), 20 ng/mL EFG (Peprotech, AF-100-15), and 2 μg/mL heparin (MP Biomedicals, 210193125) and seeded onto Matrigel-coated (Corning, 354234) plates that were previously incubated at 37°C for 2 hours (Matrigel diluted 1:100 in DMEM/F-12 with GlutaMAX Supplement [Thermo Fisher Scientific, 31331028]). After first splitting of cells, mNPCs were cultured in media conditions listed above on plates coated firstly with poly-D-Lysine (PDL) hydrobromide (Sigma, P7280) overnight followed by laminin (Roche, 11243217001) coating for 2 hours at 37°C.

### RNA-Seq.

mNPCs were harvested in TRIzol Reagent (Thermo Fisher Scientific, #15596026) and RNA isolated with Direct-zol RNA Microprep kit (Zymo, #R2060). RNA concentration and purity was measured on a Nanodrop and with Agilent RNA 6000 Nano Kit (Agilent Technologies, 5067-1511) on Agilent 2100 Bioanalyzer. All samples had RIN>9. Samples were sequenced by sequencing company Novogene (Cambridge, UK). Raw RNA-Seq data (fastq files) were downloaded from Novogene and raw reads were pseudoaligned to the mouse mm10 reference transcriptome (GRCm38) using Kallisto (version 0.48.0) (64), running the kallisto quant command with bootstrap parameter set to 100 (-b 100) for 100 bootstraps. For viewing sequencing coverage, fastq files were aligned to mm10 reference transcriptome using kallisto quant command with parameter genomebam, creating bam files that were converted to bigWig files using bamCoverage with default settings from deepTools2 (version 3.5.1) (65). Genome coverage bigwig files were viewed in the UCSC browser (66). Aligned data were imported to R (67) using tximport (version 1.30.0) (68) and mapped to mm10 genes with the BiomaRt package (69). Differential expression analysis was performed on samples containing >10 counts and genes with only one sample containing counts were excluded. We used DESeq2 (version 1.42.0) using default settings (70). To correct for false discovery rates, the z-scores returned from DESeq2 were used as an input for fdrtool with statistic=”normal” and statistical cut off was set at corrected *q* value < 0.05 (71). For further analysis we used 3 genotypes: *Kmt2a^+/+^* for wildtype samples, *Kmt2a^+/LSL^* for WDSTS samples and Nes-Cre^+/–^ *Kmt2a^+/LSL^* for assessing the in utero rescue, excluding the Nes-Cre^+/–^ *Kmt2a^+/+^* cells as they served only as another WT control. To assess the quality of the differential analysis, we performed 3 quality control checks. First, we inspected the histogram of corrected p-values (Supplemental Figure 5D), revealing an even distribution of values between 0 and 1, with a clustering of values close to 0. This suggests a calibrated differential analysis. Secondly, we observed the genotype differences on a principal component analysis (PCA) plot (Supplemental Figure 5E), after performing regularized log transformation with rlog() function on expression matrix. PCA plot reveals slight variance between biological replicates within genotypes. Lastly, an MA plot demonstrates no obvious systematic issues of the data (Supplemental Figure 5F). WebGestalt was used for ORA (72). ASD risk genes were acquired from SFARI Gene database (https://gene.sfari.org/, version Q1 2024) (38). The Complex Heatmaps package was used for generating heatmaps for gene expression visualization (version 2.18.0) (73). The expression values of differentially expressed genes were scaled to z-scores, and outliers with z-score threshold greater than 2 were excluded. IsoformSwitchAnalyzeR was used for identification of isoform switches (version 2.2.0) (74). Venn diagrams were created with DeepVenn (75) and Fisher’s test was applied for statistical analysis using GeneOverlap (76).

### CUT&RUN.

CUT&RUN was performed according to CUTANA CUT&RUN Protocol (v.1.7 EpiCypher) from 160,000 cells per sample, with *n* = 3 *Kmt2a^+/+^*, *n* = 4 *Kmt2a^+/LSL^*, and *n* = 4 Nes-Cre^+/–^ *Kmt2a^+/LSL^*. Cells were permeabilized with 0.01% digitonin buffer (Sigma, D141) and *E. Coli* DNA (EpiCypher, 23618-1401) was used as spike-in for normalization (0.2 ng final concentration per sample). The antibodies used were Rabbit IgG Antibody (EpiCypher, 23613-0042, 0.5μL per sample) and H3K4me1 (Abcam, ab8895, 0.25μL per sample). DNA was isolated using Genomic DNA Clean & Concentrator (Zymo, D4065). Library preparation was done using TruSeq RNA CD Index plate (Illumina, 20019792). Quality of samples were assessed using Agilent Bioanalyzer DNA high sensitivity kit (Agilent Technologies, 5067-4626). DNA samples were pooled to a 1.8 pM concentration and sequenced on a NextSeq 550Dx instrument (Illumina) using NextSeq 500/550 High output kit v2.5 (Illumina, 20024907). Demultiplexing was performed using Illumina bcl2fastq command with the options --ignore-missing-control --ignore-missing-positions --ignore-missing-filter. Fastq sample files were aligned to the mouse mm10 genome using Bowtie2 (version 2.4.4) (77) for paired end reading. Aligned sam files were sorted, indexed and converted to bam files using SAMtools (version 1.17) (78). For visualizing H3K4me1 peaks in UCSC, bam files for all samples for each genotype were merged with SAMtools. They were then converted to bigwig files with deepTools (version 3.5.1) and normalized to 1x mouse genome with --normalizeUsing RPGC (65). Reads were merged for each genotype, and these merged files were used for peak calling against the merged IgG samples for each genotype using MACS3 (version 3.0.0) with settings -q 0.1, -maxgap-300, -keep-dup all (79). First, the raw reads for each sample were counted using dba.count() function from Diffbind (version 3.12.0) with “score= DBA_SCORE_READS”, followed up with retrieving the matrix with dba.peakset() function, with settings “bRetrieve=TRUE” (80). Raw count matrix was filtered to only include peaks where the count was greater than 10 for at least one genotype. Count matrix was used as input for DESeq2 where differential analysis was performed. FDRtool with statistic=“normal” was applied to correct for false discovery rates, with a cut off at corrected *P* < 0.001. H3K4me1 peaks were annotated to nearest gene using annotatePeak() function from ChIPseeker package (version 1.5.1) (81). Mouse NPC and Neuronal enhancer sites were retrieved from Enhancer Atlas (version 2.0) (39). Heatmaps and plotprofiles were plotted using deepTools2 (version 3.5.1) (65).

### Genome-wide sequencing (GWS).

DNA from mNPCs in culture were harvested from *Kmt2a^+/LSL^* primary hippocampal cell line sample described above (1 million cells) using Monarch® HMW DNA Extraction Kit (NEB, T3050). We sheared the high molecular weight (HMW) DNA to achieve consistent and narrow size ranges to 20kb using the Megaruptor 3 Shearing kit (E07010003, Diagenode). Megaruptor 3 was performed using customized settings, including a concentration of 50 ng/μL, a volume of 70 μL, and a speed of 30. Subsequently, we measured the sheared DNA size using the HS Large Fragment 50 kb kit (DNF-464-0500, Agilent) on a Fragment Analyzer and analyzed by the High Sensitivity Large Fragment 50 kb Analysis software. We performed library preparation with a Ligation Sequencing kit V14 (SQK-LSK114, ONT), and sequenced by loading the sample into an R10.4.1 flow cell (FLO-PRO114M, ONT) loaded on a PromethION 24 sequencer based on the manufacture’s guidelines (ONT). Nanopore sequencing was conducted using customized run settings, which included a Run Limit of 96 hours, a Minimum Read Length of 200 bp, and Super-accurate Base calling. To optimize output, we washed the flow cell once after 22 hours of sequencing using the Flow Cell Wash kit XL (EXP-WAS004-XL, ONT), followed by a 1.5-hour wait period and flushing of the flow cell with Flow Cell Priming Mix twice by using Sequencing Auxiliary Vials V14 (EXP-AUX003, ONT). We utilized the remaining DNA library from the previous preparation for loading. Data acquisition and base calling in real-time using PromethION device and MinKNOW software developed by ONT. Nanopore sequencing yielded approximately 86.43 Gb of estimated bases with an average read length of 23.5 kb. All fastq files were merged into 1 fastq file using cat command in Linux. A sam file was created by aligning the fastq file to the mouse genome using minimap2 with –map-ont function for ONT data (82) and reference genome mm10. Sam file was converted to a bam file, sorted and indexed using samtools (78). Sequencing results were viewed in IGV (83).

### Statistics.

Data was analyzed using Prism 9 (v 9.4.1, GraphPad Software, Boston Massachusetts USA) and R (67). Unless otherwise stated, for comparisons between 2 groups, significance was calculated with a 2-tailed unpaired *t* test. For comparisons in which WT and *Kmt2a^+/LSL^* groups were each tested against the rescue group, significance was calculated using a 1-way ANOVA with Dunnett’s correction for multiple comparisons. When these comparisons involved multiple stainings or layers within the same analysis, a 2-way ANOVA with Dunnett’s correction for multiple comparisons was used. A *P* value less than 0.05 was considered significant.

### Study approval.

Import of mice and experiment protocols were in accordance with Icelandic Food and Veterinary Authority (license no. 2208602) and accepted by National Expert Advisory Board on Animal Welfare.

### Data availability.

All data are available in the main text or the supplementary materials. The RNA-Seq and CUT&RUN data associated with this study are available at the Gene Expression Omnibus (GEO) under accession nos. GSE271275 and GSE271388. ONT sequencing of the *Kmt2a^+/LSL^* model is available in the SRA under the accession no. SRR33583431. Values for all data points in graphs are reported in the Supporting Data Values file.

## Author contributions

Conceptualization was contributed by HTB. Methodology was contributed by HTB, TR, KJA, KM, JO, AOS, VBD, and ARZ. Formal analysis was contributed by TR, KJA, KM, SP, VBD, AB, and KPF. Investigation was contributed by TR, KJA, KM, AOS, JO, and ARZ. Data curation was contributed by TR, KJA, KM, SP, VBD, AB, and KPF. Validation was contributed by TR, KJA, and ARZ. Visualization was contributed by TR, KJA, KM, and VBD. Funding acquisition was contributed by HTB and CML Resources were contributed by HTB. Project administration was contributed by TR, HTB, and KJA. Supervision was contributed by HTB. Writing of the original draft was contributed by TR. Review and editing were contributed by HTB, TR, and KJA.

## Funding support

This work is the result of NIH funding, in whole or in part, and is subject to the NIH Public Access Policy. Through acceptance of this federal funding, the NIH has been given a right to make the work publicly available in PubMed Central.

The Eimskip University Fund, UI doctoral grant (TR and JO)

The Rannis, Icelandic Research Fund, Grant of Excellence 217988-051 (HTB and JO), and postdoctoral grant 2410204-051 (KM)

The Wiedemann-Steiner Foundation, project grant (HTB)

The NIH Jackson Laboratory Precision Genetics Grant U540D030157 (CML).

## Supplementary Material

Supplemental data

Unedited blot and gel images

Supplemental table 1

Supplemental table 2

Supplemental table 3

Supporting data values

## Figures and Tables

**Table 1:**
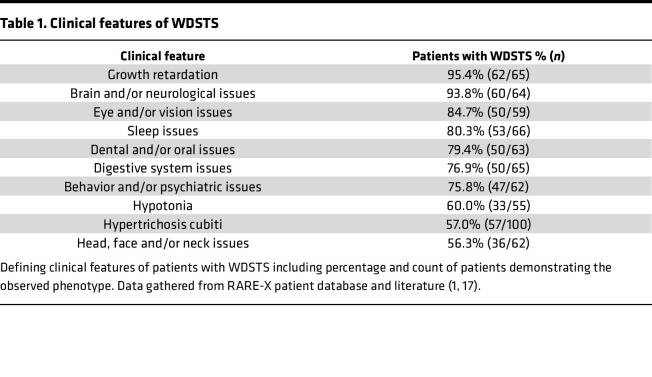
Clinical features of WDSTS

## Version 1 09/16/2025

In-Press Preview

## Version 2 10/22/2025

Electronic publication
