# Supplementary material for: In utero rescue of neurological dysfunction in a mouse model of Wiedemann-Steiner syndrome
Source: JCI Insight. 2025 Sep 16;10(20):e187039. doi: 10.1172/jci.insight.187039 (PMC12581663; doi:10.1172/jci.insight.187039)
Supplement: Supplemental data [file jciinsight-10-187039-s089.pdf]

Supplementary Data: *In-utero* rescue of neurological dysfunction in a mouse model of Wiedemann-Steiner syndrome

Supplementary Methods

RNA isolation from brain and Real Time Quantitative Polymerase Chain Reaction (RT-qPCR)

Total RNA was isolated from whole brain tissue of P1 pups using TRIzol™ Reagent (Thermo Fisher Scientific, #15596026) according to the manufacturer's instructions. RNA concentration was measured on NanoDrop (Thermo Fisher Scientific), and 0.5 ng of RNA was used for input for cDNA synthesis using High-Capacity cDNA Reverse Transcription Kit (Applied Biosystems, #4368814), run on the MiniAmp Thermal Cycler (Applied Biosystems). Luna Universal qPCR Master Mix (NEB, M3003) was used for RT-qPCR reactions and run on CFX384 Real-Time PCR Detection System (Bio-Rad). Each biological replicate of the RT-qPCR assay in this study was carried out in technical triplicates.

Histology

Samples were taken from the belly and backs of newborn pups, fixed in 4% paraformaldehyde, and then processed at the Department of Pathology, Landspitali National University Hospital, Reykjavik, Iceland. The tissue samples were paraffin-embedded and cut into 1.5µm sections for hematoxylin and eosin (H&E) staining and 3µm sections for immunohistochemistry (IHC). H&E staining was done using standard methods. Sections for immunohistochemistry were de-paraffinized and rehydrated in xylene and ethanol. Antigen retrieval was done with Envision Flex Target Retrieval Solution, high 9 (Agilent, K800421-2). Sections were immunostained in AutostainerLink 48 from Agilent with EnVision Detection System Peroxidase/DAB, Rabbit/Mouse kit (Agilent, K4065). Incubation with primary antibody Calretinin (Agilent, M7245) was performed at room temperature for 30 min. After incubation with a primary antibody, sections were incubated with EnVision FLEX/HRP. Sections were washed between steps with Tris-buffered NaCl solution with Polysorbate 20 pH 7.6 (Agilent, S3306). All sections were incubated with 3,3′-diaminobenzidine solution (Agilent, K4065) for 10 min. Sections were counterstained with hematoxylin for 30 seconds. Finally, sections were dehydrated with 100% ethanol and xylene followed

by cover slipping with mounting medium (Pertex, Histolab). Images were acquired with NanoZoomer XR (HAMAMATSU) scanner. Sections were analyzed using NDP view (version 2.7.25). Hair follicles were counted on 8 mm of skin, each section was counted 4 times, and the average number was plotted per mm of skin.

CBC measurement and hair removal

Blood samples for Complete Blood Count (CBC) were collected in EDTA blood tubes from 3-5 m/o mice (n=8 per genotype) and measured on VetScan HM5 v2.1 (Abaxis Inc, USA). Hair was removed from the backs of adult mice using Veet Hair Removal Crème (Reckitt Benckiser).

Supplemental Figure 1

A

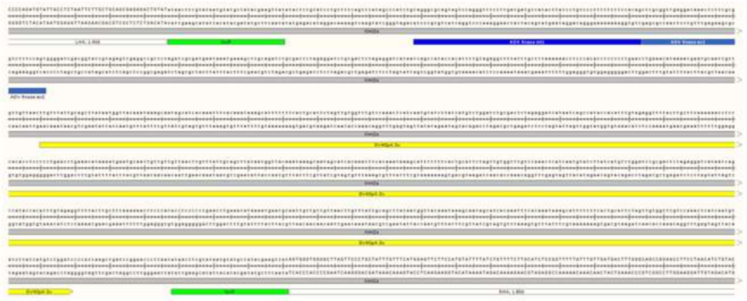

B

crRNA	Sequence	PAM	Score	Locus	Gene
Kmt2a_in1_crRNA1:	CTGCAGCGAGAGACTGTATG	AGG	100.0	Chr9: - 44873762	*Kmt2a*
CTGCAGCGAGAGACTGTATG	TTGGAGCCAGAGACTGTATG	AAG	2.8	Chr 3: +60581077	*Mbnl1*
Kmt2a_in1_crRNA2:	GAGACTGTATGAGGTATCAG	TGG	100.0	Chr9: 44873753	*Kmt2a*
GAGACTGTATGAGGTATCAG	AAGACTGTATGAAGTATCAG	AAG	3.9	Chr4: -59081019	*Shoc1*

C D

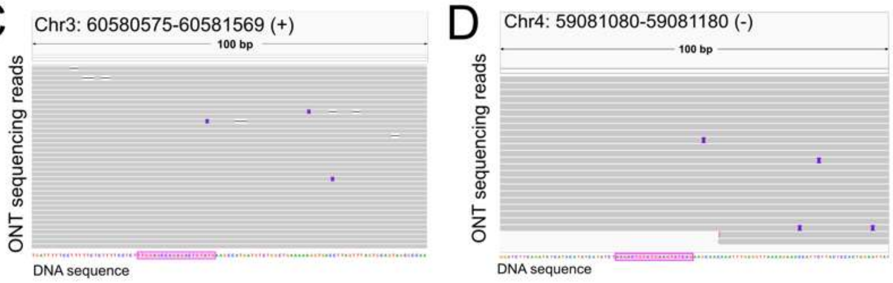

E

	Het x Het			
35724	WT	Het	Hom	
Female	8	20	0	
Male	14	12	0	
Total	22	32	0	54
%	40.7	59.3	0.0	

F

	Het x Het			
35725	WT	Het	Hom	
Female	11	22	0	
Male	13	13	0	
Total	24	35	0	59
%	40.7	59.3	0.0	

G

	Het x Het			
35727	wt	het	Hom	
female	6	10	2	
male	3	11	3	
total	9	21	5	35
%	25.7	60.0	14.3	p, ns

Supplemental Figure 1: (**A**) DNA sequence of loxP-stop-loxP cassette insertion in intron 1 of *Kmt2a* out listed by Jackson Laboratory. (**B**) Table lists DNA sequence of CRISPR RNA (crRNA) for loxP-stop-loxP cassette insertion using CRISPR/Cas9, the DNA sequence targeted by the crRNA, their PAM site, off targeting score and location. Off-target scoring was performed by Jackson Laboratory, with higher off-target editing risk set at score>20.0 for unlinked non-canonical PAM (NAG) in coding region. Oxford Nanopore Technologies (ONT) sequencing results of potential CRISPR off-target sites viewed in Integrative Genomics Viewer (IGV) showing no off-target effects for (**C**) chromosome 3 and (**D**) chromosome 4. Het-het breeding ratio of *Kmt2a*$^{+/LSL}$ mice, (**E**) stock #35724 C57BL/6J-*Kmt2a*^{em7Lutzy}/J and (**F**) stock #37525 C57BL/6J-*Kmt2a*^{em8Lutzy}/J, demonstrating no homozygous pups born, confirming the model's lethality in homozygosity. (**G**) Breeding ratio of post-Cre het-het mice (Stock #35727 C57BL/6J-*Kmt2a*$^{em7.1Lutzy}$/J), showing Sox2-Cre Tg is not segregating in these mice.

Supplemental Figure 2

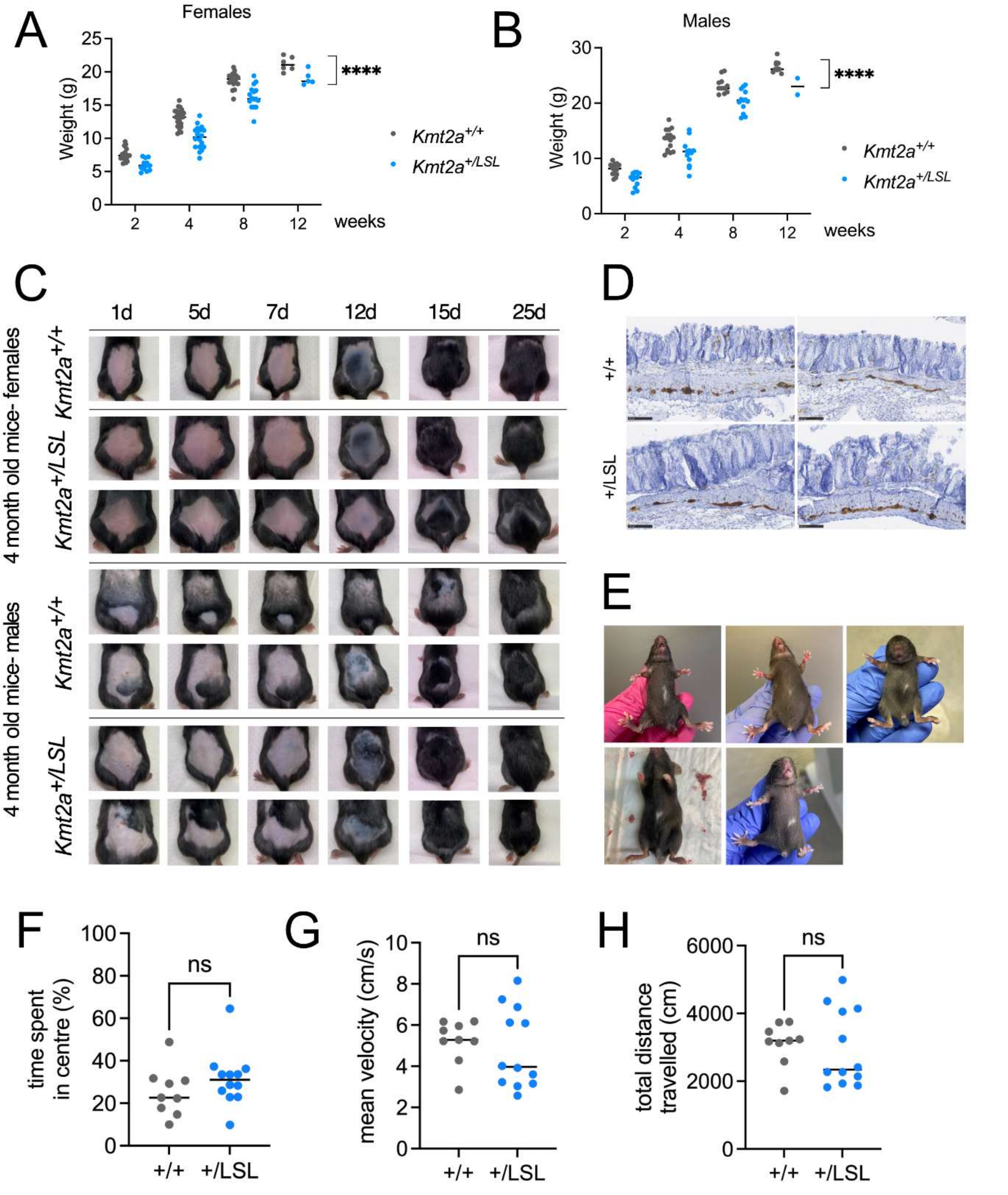

Supplemental Figure 2: Weight data from *Kmt2a*$^{+/+}$ and *Kmt2a*$^{+/LSL}$ mice at 2, 4, 8 and 12 weeks for (**A**) females and (**B**) males. (**C**) Hair regrowth after hair removal for 4-month-old mice (**D**) Calretinin staining of the distal colon of *Kmt2a*$^{+/+}$ and *Kmt2a*$^{+/LSL}$ P0 pups. Both genotypes show positive calretinin staining (brown). Scale bar is 100µm. (**E**) Examples of white belly spots present in *Kmt2a*$^{+/LSL}$ mice. 8-week-old *Kmt2a*$^{+/LSL}$ mice do not show a significant difference in the percentage of time spent in center (**F**), the mean velocity recorded (**G**), nor the total distance travelled (**H**) in an open field test. ns: not significant, *p<0.05, **p<0.01, ***p<0.001, ****p<0.0001.

Supplemental Figure 3

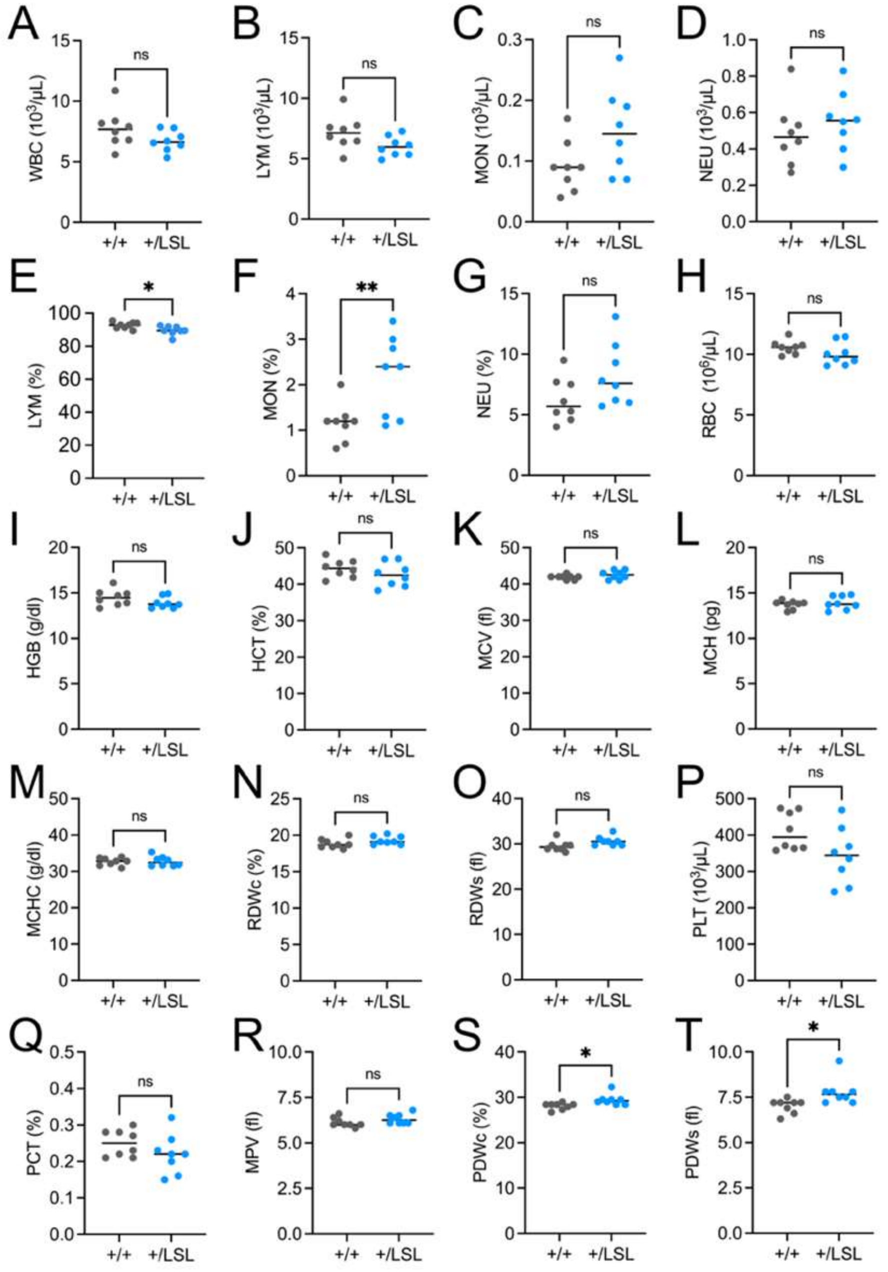

Supplemental Figure 3: Blood samles from 3-5 m/o mice showing (**A**) WBC - Total white blood cell count (10^3/μl) (**B**) LYM - lymphocyte count (10^3/μl) (**C**) MON - monocyte count (10^3/μl) (**D**) NEU - neutrophil count (10^3/μl) (**E**) LYM% — lymphocyte percentage (%) (**F**) MON% — monocyte percentage (%) (**G**) NEU (%) – neutrophil percentage (%) (**H**) RBC — red blood cell count (10^6/μl) (**I**) HGB — hemoglobin (g/dl) (**J**) HCT — hematocrit (%) (**K**) MCV — mean corpuscular volume (fl) (**L**) MCH — mean corpuscular hemoglobin (pg) (**M**) MCHC — mean corpuscular hemoglobin concentration (g/dl) (**N**) RDWc, red cell distribution width, coefficient of variation (%) (**O**) RDWs red cell distribution width, std dev (fl) (**P**) PLT — platelet count (10^3/μl) (**Q**) PCT — platelet crit (%) (**R**) MPV — mean platelet volume (fl) (**S**) PDWc, — platelet distribution width, coefficient of variation (%) (**T**) PDWs, platelet distribution width, std dev (fl). ns: not significant, *$p<0.05$, **$p<0.01$, ***$p<0.001$, ****$p<0.0001$.

Supplemental Figure 4

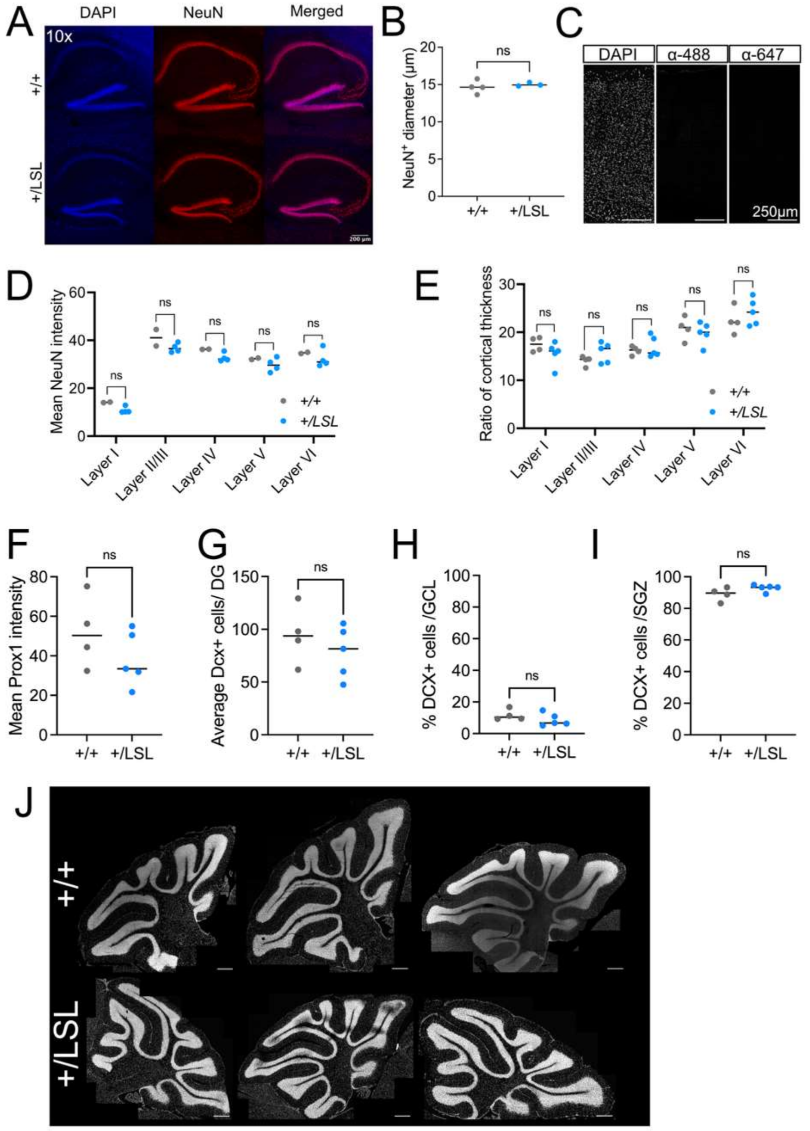

Supplemental Figure 4: Immunostainings of 9-week-old mouse brains. (**A**) Representative figures from NeuN staining. The scale bar is 200µm. (**B**) Quantification of NeuN+ nuclei size from the granule cell layer of the dentate gyrus of the hippocampus of *Kmt2a*$^{+/+}$ and *Kmt2a*$^{+/LSL}$ mice. (**C**) 2°AB-only control of cortical stainings with NeuN (488) and Tbr-1 (647) (**D**) Mean NeuN intensity in the layers of the cortex for *Kmt2a*$^{+/+}$ and *Kmt2a*$^{+/LSL}$ mice. (**E**) The thickness ratio (in percentages) for each cortical layer for each genotype. (**F**) Mean Prox1 intensity in the DG of the hippocampus for *Kmt2a*$^{+/+}$ and *Kmt2a*$^{+/LSL}$ samples. (**G**) Quantification of Dcx positive cells (Dcx+) in the DG of the hippocampus compared between genotypes. (**H**) Percentage of Dcx+ cells found in the granule cell layer (GCL) of the DG. (**I**) Percentage of Dcx+ cells found in the subgranular zone of the DG. (**J**) Representative images of the cerebellum of *Kmt2a*$^{+/+}$ and *Kmt2a*$^{+/LSL}$ mice. ns: not significant, *p<0.05, **p<0.01, ***p<0.001, ****p<0.0001.

Supplemental Figure 5

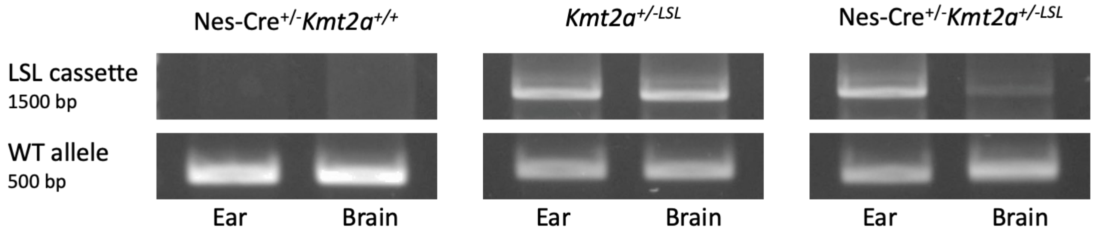

Supplemental Figure 5. PCR products from gDNA samples from Nes-Cre$^{+/-}$*Kmt2a*$^{+/+}$, *Kmt2a*$^{+/LSL}$ and Nes-Cre$^{+/-}$*Kmt2a*$^{+/LSL}$ mice run on an agarose gel, comparing visually the presence of LSL cassette in the nervous system (hippocampus) and outside the nervous system (ear clips). There is removal of the LSL cassette in the Nes-Cre$^{+/-}$*Kmt2a*$^{+/LSL}$ mice.

Supplemental Figure 6

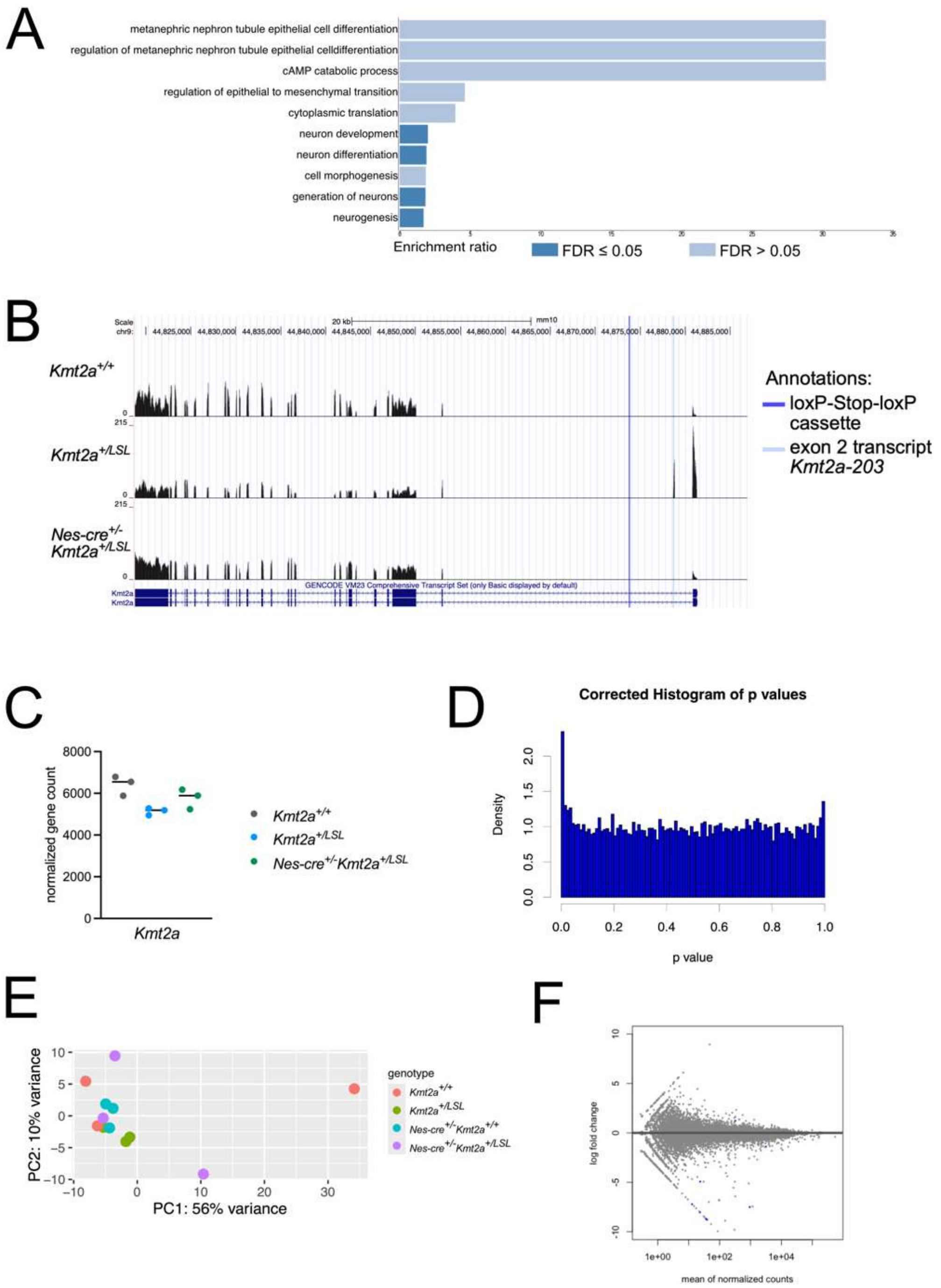

Supplemental Figure 6: (**A**) Over representation analysis of the top decile rescued genes. Figure shows pathways enriched in dark blue (with FDR<0.05) (**B**) UCSC browser tracks of genome coverage of RNA seq files showing a decrease of *Kmt2a* coverage in *Kmt2a*$^{+/LSL}$ cells for full length transcripts. Only in *Kmt2a*$^{+/LSL}$ cells is there a presence of a peak mapped to exon 2 of the short *Kmt2a-203* transcript. (**C**) Normalized gene count values for *Kmt2a* from RNA seq data. (**D**) Histogram of corrected p-values from differential expression analysis of RNA seq data. (**E**) PCA plot of the expression matrix of all genotypes (F) MA plot of log2 fold change values plotted against mean of normalized counts.

Supplemental Figure 7

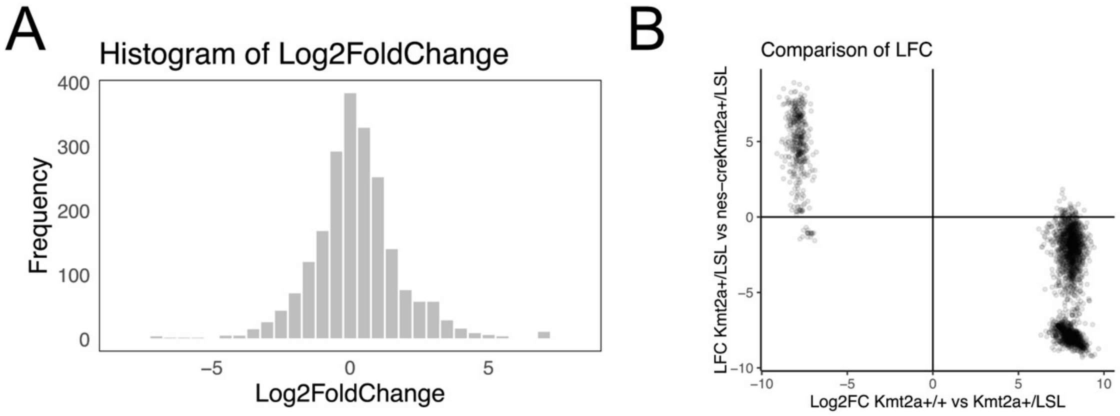

Supplemental Figure 7: (**A**) Histogram of $\log_2$ fold changes for gene expression of genes linked to neuronal enhancers. (**B**) Correlation plot showing the correlation between H3K4me1 changes between *Kmt2a*$^{+/+}$ vs *Kmt2a*$^{+/LSL}$ and then between *Kmt2a*$^{+/LSL}$ vs Nes-Cre$^{+/-}$*Kmt2a*$^{+/LSL}$ contrasts.

Supplemental Figure 8

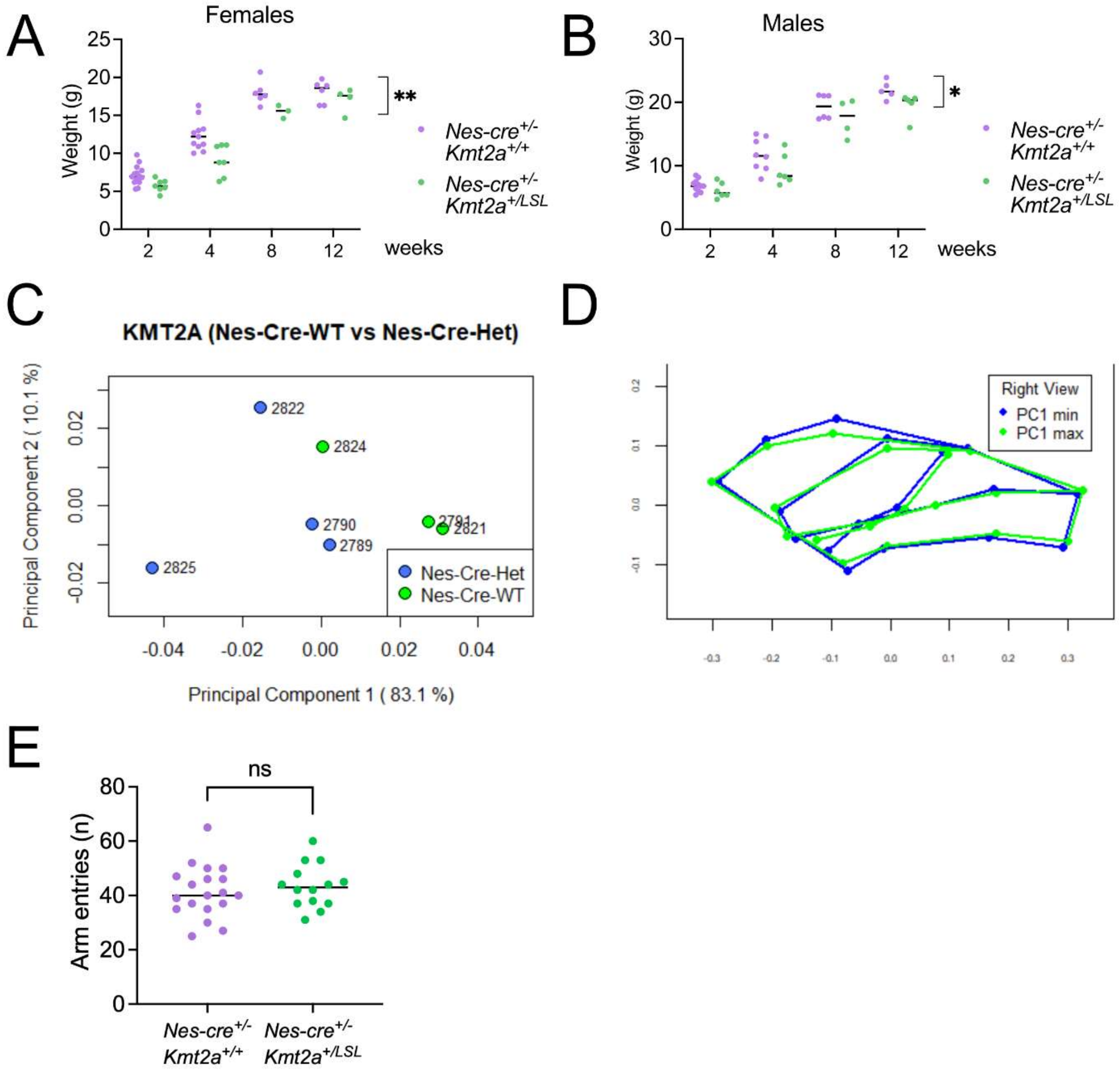

Supplemental Figure 8: Weight data from Nes-Cre$^{+/-}$*Kmt2a*$^{+/LSL}$ and Nes-Cre$^{+/-}$*Kmt2a*$^{+/+}$ (**A**) female (mixed effects model, p<0.01) and (**B**) male mice (mixed effects model, p<0.05). (**C**) PCA of craniofacial abnormalities of Nes-Cre$^{+/-}$-*Kmt2a*$^{+/LSL}$ vs. Nes-Cre$^{+/-}$-*Kmt2a*$^{+/+}$ shows separation of genotypes along PC1. (**D**) Similar to the analysis of the *Kmt2a* model (Fig. 1E-H), the Nes-Cre$^{+/-}$*Kmt2a*$^{+/LSL}$ mice (2-3.5 month old) are positioned at the negative end of PC1 and characterized by increased height and width of the neurocranium, a shortened midface, and ventral bowing of the cranium.(**E**) Comparison of total arm entries in a Y-maze between 8-10 week old Nes-Cre$^{+/-}$*Kmt2a*$^{+/LSL}$ mice and their wild-type littermates

Supplemental Table 4. WDSTS clinical features and their presence in WDSTS patients and mouse model.

Clinical feature	WDSTS patients % (n)	Presence in mouse model
Growth retardation	95.4% (62/65)	present
Brain and/or neurological issues	93.8% (60/64)	present
Eye and/or vision issues	84.7% (50/59)	unknown
Sleep issues	80.3% (53/66)	unknown
Dental and/or oral issues	79.4% (50/63)	unknown
Digestive system issues	76.9% (50/65)	unknown
Behavior and/or psychiatric issues	75.8% (47/62)	present
Hypotonia	60.0% (33/55)	present
Hypertrichosis cubiti	57.0% (57/100)	present
Head, face and/or neck issues	56.3% (36/62)	present
Ear and/or hearing issues	44.3% (27/61)	unknown
Lungs and/or breathing issues	40.0% (26/65)	unknown
Issues with bone, cartilage and/or connective tissue	39.0% (23/59)	present
Hormones and/or hormone-producing glands issues	33.3% (19/57)	unknown
Kidney, bladder and/or genital issues	31.1% (19/61)	unknown
Immune system issues	26.3% (15/57)	unknown
Skin issues	24.6% (15/61)	unknown
Hear and/or blood vessel system issues	23.8% (15/63)	unknown
Blood or bleeding issues	1.6% (1/63)	present

Supplemental Table 5. Landmarks collected for analysis of CT scans.

**Midline**	
nal	nasale
ids	interdentale superior
pns	posterior nasal spine
nas	nasion
brg	bregma
lam	lambda
int	interparietal
opi	opisthion
pcg	posterior crista galli
sem	sphenoethmoid synchondrosis
pbm	intrasphenoid synchondrosis
bom	sphenooccipital synchondrosis
bas	basion
**Bilateral**	
rnot,lnot	infraorbital notch
rmjs,lmjs	maxillojugal superior
rjss,ljss	jugosquamosal superior
rfsc,lfsc	frontosphenoid crest
ream,leam	external auditory meatus
rast,last	asterion
rpmm,lpmm	premaxillo-maxillary suture
rptg,lptg	pterygoid hamulus
rfmc,lfmc	condyle at foramen magnum
