# Supplementary figures and images for: In utero rescue of neurological dysfunction in a mouse model of Wiedemann-Steiner syndrome
Source: JCI Insight. 2025 Sep 16;10(20):e187039. doi: 10.1172/jci.insight.187039 (PMC12581663; doi:10.1172/jci.insight.187039)

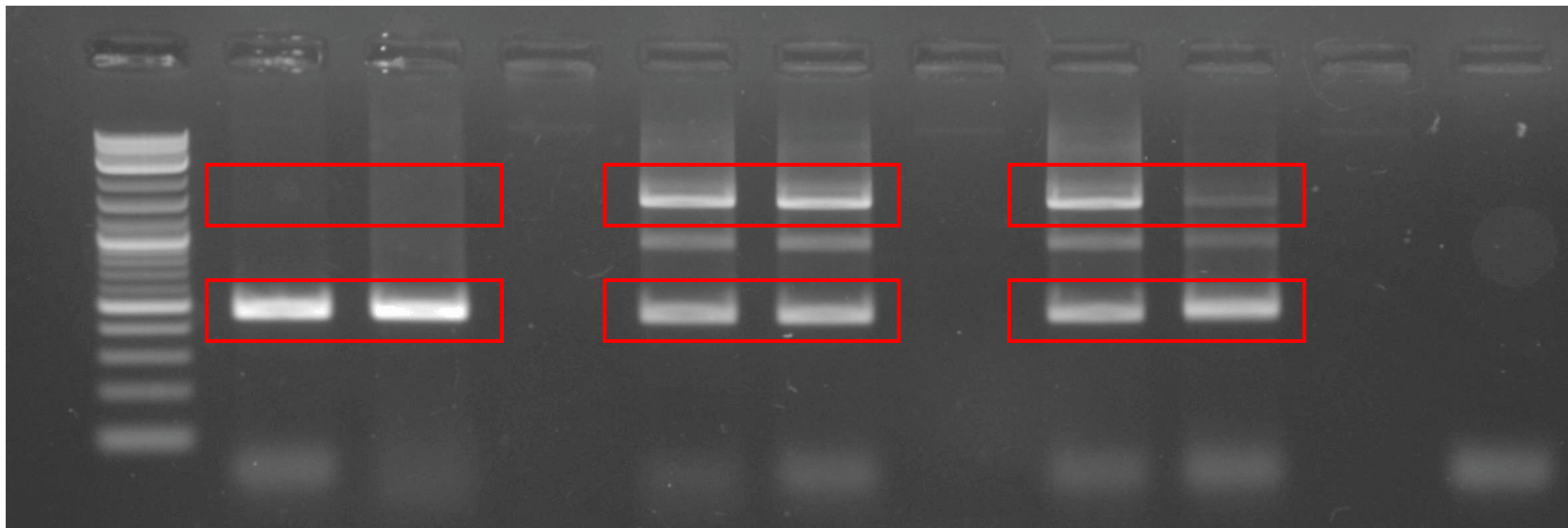

Supplement: Unedited blot and gel images [file jciinsight-10-187039-s090.pdf]
